# Intensive home visiting for adolescent mothers in the Family Nurse Partnership in England 2010–2019: a population-based data linkage cohort study using propensity score matching

**DOI:** 10.1136/bmjph-2023-000514

**Published:** 2024-02-20

**Authors:** Francesca Cavallaro, Ruth Gilbert, Jan Van der Meulen, Sally Kendall, Eilis Kennedy, Katie Harron

**Affiliations:** 1 UCL Great Ormond Street Institute of Child Health, London, UK; 2 London School of Hygiene & Tropical Medicine, London, UK; 3 University of Kent, Kent, UK; 4 Tavistock and Portman NHS Foundation Trust, London, UK; 5 UCL Great Ormond Street Institute of Child Health, UCL, London, UK

**Keywords:** Public Health, Confounding Factors, Epidemiologic, Secondary Prevention

## Abstract

**Background:**

We evaluated the effectiveness of the Family Nurse Partnership (FNP), an intensive home visiting programme aiming to improve birth outcomes, child health and development, and to promote economic self-sufficiency among teenage mothers.

**Methods:**

We created a linked cohort of >130 000 mothers aged 13–19 years with live births between April 2010 and March 2019, using administrative data from health (Hospital Episode Statistics), education and children’s social care (National Pupil Database). Using propensity score matching, we compared indicators of child maltreatment, health and development outcomes, and maternal hospital utilisation and educational outcomes up to 7 years following birth for mothers who did or did not enrol in FNP.

**Results:**

We found no evidence of an association between FNP and indicators of child maltreatment, except for an increased rate of unplanned admissions for maltreatment/injury-related diagnoses up to age 2 years for children born to FNP mothers (6.6% vs 5.7%, relative risk (RR) 1.15; 95% CI 1.07, 1.24). There was weak evidence that children born to FNP mothers were more likely to achieve a good level of development at age 5 years (57.5% vs 55.4%, RR 1.05; 95% CI 1.00, 1.09). FNP mothers were less likely to have a subsequent delivery within 18 months of the index birth (8.4% vs 9.3%, RR 0.92; 95% CI 0.88, 0.97).

**Conclusion:**

Our study supports findings from previous evaluations of FNP showing no evidence of benefit for child maltreatment or health outcomes measured in administrative data. Bias by indication, and variation in the intervention and usual care, may have limited our ability to detect effects. Future evaluations should capture more information on maternal risk factors and additional outcomes relating to maternal/child well-being.

**WHAT IS ALREADY KNOWN ON THIS TOPIC**

**WHAT THIS STUDY ADDS**

- This population-based cohort study examined data for >30 000 mothers participating in the FNP in England over a 9-year period and is the largest evaluation of the FNP in the UK to date.
- Our findings show little evidence of impact on indicators of child maltreatment, but some weak evidence of improvements in child development measures and reductions in rapid repeat pregnancies.
- As we could only control for the maternal risk factors associated with enrolment in FNP recorded in administrative data, residual confounding could have limited our ability to detect beneficial effects of the programme.

**HOW THIS STUDY MIGHT AFFECT RESEARCH, PRACTICE OR POLICY**

- More research is needed to understand which elements of intensive interventions are most effective, for whom and when, and to help inform decisions about whether it is better to commission highly intensive services for a small portion of the target population, or to extend and enhance universal services to better support all adolescent mothers.

## Introduction

 Each year, approximately 3% of babies (~16 000 in 2020) are born to mothers aged less than 20 years in England and Wales.^1^ Adolescent mothers are more likely to experience adversity, be less engaged with education and employment, and have rapid repeat pregnancies compared with older mothers.^2^,^5^ For their children, young maternal age is associated with higher incidence of preterm birth and low birth weight,^6 7^ and a greater risk of child maltreatment and associated adverse long-term consequences including poorer physical health, social, emotional and cognitive outcomes.^8^,^10^ These adverse maternal and child outcomes of adolescent pregnancy, associated with social adversity, disruption to education and employment, and child-rearing practices, are of major importance to public health research and the National Health Service (NHS).^11 12^ Understanding how best to target services to the most vulnerable mothers is key to improving health for these mothers and their children.

Many intensive health services aiming to reduce maltreatment and/or inequalities between adolescent and older mothers have been trialled. While some meta-analyses have found positive effects across a range of child and parent outcomes including maltreatment,^13^,^15^ others have found a more ‘gloomy’ picture,^16^ in part due to the effectiveness of different components of these programmes for different outcomes.^17 18^ One programme consistently recognised for its effects is the Family Nurse Partnership (FNP),^19^ which is currently the only programme recommended within the UK government Healthy Child Programme, and which has been commissioned in >130 English Local Authorities since 2007. Mothers enrolled in the FNP receive up to 64 home visits by a dedicated family nurse, from early pregnancy until the child’s second birthday. The FNP aims to improve birth outcomes, child health and development, and to promote economic self-sufficiency among young mothers.^20^

Most of the evidence of effectiveness of the FNP stems from three randomised trials of the Nurse-Family Partnership (NFP) conducted in the USA, which evaluated a wide range of maternal and child outcomes, with up to 20 years of follow-up. The three USA trials showed mixed but overall positive impacts on child health and development outcomes, and on some maternal outcomes. A more recent Netherlands randomised controlled trial (VoorZorg, enrolling in 2007–2009) also reported a reduction in child abuse/maltreatment reports by age 3 years in the FNP arm. These results contrast with a more recent trial of 5670 Medicaid-eligible nulliparous pregnant mothers recruited between 2016 and 2020 in South Carolina, which found no evidence of an effect on birth outcomes (preterm birth, low birth weight, small for gestational age and perinatal death), and the Building Blocks trial of FNP in England (enrolling approximately 1600 expectant mothers in 2009–2010), which showed no evidence of impact of FNP on most child outcomes, with the exception of some cognitive outcomes including maternally reported child cognitive and language development in the first 2 years of life, and a good level of development at school entry (a measure of school readiness at age 5 years).^21^

Historically, FNP has been delivered in a similar way in England as the NFP is delivered in the USA (although more flexibility has been introduced in recent years).^22^ The licensing agreement stipulated that sites should follow a number of core model elements, so that the FNP could be replicated consistently, in order for the conditions upon which the previous evidence from the USA were based to be replicated. However, there are notable differences in eligibility criteria. Therefore, two potential explanations for different results in England compared with the USA are variation in usual care and in eligibility. First, the social safety net is likely to be stronger in England than in the USA, with better access to services for adolescent mothers not enrolled in FNP (including the minimum five mandated health visiting contacts, universal healthcare free at the point of care, services provided through free children’s centres, etc), which may explain the lack of association for most outcomes in England. Second, the main eligibility criterion for enrolment in FNP in England is maternal age: adolescents who are aged up to 19 years at last menstrual period and who are first-time mothers are eligible for enrolment. In contrast, additional socioeconomic criteria such as unemployment, low educational level or low income are used in combination with maternal age in other countries. As a result, the population of young mothers enrolled in trials in other countries is a more selected and vulnerable group than in England, and may therefore stand to benefit more from the FNP (as evidenced by greater effectiveness in socioeconomically deprived groups demonstrated in the US trials).

Despite the results of the Building Blocks trial, there remains strong support for the programme locally.^2023^,^25^ Around one in four eligible mothers are enrolled in the FNP within Local Authorities that offer the programme; mothers who are not offered the FNP, or who decline, are offered usual care for adolescent mothers, which varies locally.^26 27^ Generating evidence on which groups of mothers and their children benefit from the real-world implementation of the FNP in England is therefore needed to help inform targeting and commissioning of services, especially in the context of findings from the US trials which suggest that the youngest, most disadvantaged mothers are likely to benefit most from FNP.^24^ This evidence is being called for by service providers who need to understand the value of interventions in the context of their target populations and local services, in order to inform commissioning and justify spending.^28^ Furthermore, usual care available to adolescent mothers is likely to have declined between the Building Blocks trial study period and after the introduction of austerity measures in England—in particular, health visitor budgets have decreased since responsibility for commissioning of health visiting services shifted from the NHS to local government in England in 2015.^29^

Linkage of existing administrative records provides a cost-efficient means of evaluating services as they are implemented in the real world, overcoming some of the constraints of randomised trials.^30^ Our population-based study used longitudinal linked observational data between the health, education and social care sectors for all mothers enrolled in the FNP in England since 2010, to evaluate the effects of the intervention on outcomes of eligible mothers and their children up to age 7 years. We aimed to generate evidence on which groups of mothers and children benefit from the real-world implementation of FNP in England in order to inform the targeting and commissioning of services.

## Methods

### Data sources and linkage

We used linked hospital records from Hospital Episode Statistics (HES), education and social care records from the National Pupil Database (NPD) and FNP programme data (from the FNP Information System) for mothers and their children.

HES is a data warehouse containing details of all hospital admissions (from 1997), outpatient appointments (from 2003), and Accident and Emergency (A&E) visits (from 2010) at NHS hospitals in England.^31^ In addition to the birth record, we linked information from hospital admissions and A&E attendances for mother and child (including up to 5 years before delivery for the mother; see online supplemental figure 1).

NPD includes information on pupils attending state schools or children in contact with social care services in England. Data on assessments, attainment and progression at each Key Stage were extracted, alongside information on free school meals (FSM), special educational needs (SEN) provision, and absences and exclusions.^32^ We also used information from the Early Years Census and Early Years Foundation Stage Profile (EYFSP). These data include whether the child achieved a good level of development at school entry (age 5 years; if children are at the expected level for the 12 early learning goals within the five areas of learning relating to: communication and language; personal, social and emotional development; physical development; literacy; and mathematics), which we used as a proxy for school readiness, as well as Key Stage 1 assessment data (formal teacher assessments at age 7 years).

Linkage between the FNP Information System and HES was conducted using deterministic linkage by NHS Digital (98.5% of FNP mothers were linked to an HES record); linkage with the NPD was conducted by the Department for Education using a matching algorithm requiring agreement (full or ‘fuzzy’) on names, date of birth and postcode (84.1% of mothers were linked to an NPD record). Our approach built on previous linkage of education and health records, and validated methods of linking hospital records for mothers and babies.^33^,^35^

### Study population

Our study population included all first-time mothers aged 13–19 years at last menstrual period with live births in England between 1 April 2010 and 31 March 2019 within the 136 of 152 Local Authorities in England that had an active FNP site between 2010 and 2019.^36^ We identified mothers who had participated in the FNP from the FNP programme data. Our comparison group of eligible mothers included all other mothers in our study cohort; this includes eligible mothers who were not offered the FNP (as there are not enough places for all eligible mothers) and those who were offered a place but declined to enrol (we were not able to distinguish between these two groups).

### Outcomes

We selected outcomes for the FNP evaluation based on the FNP logic model (table 1).^37^ Derivation of these outcomes is described in detail in online supplemental tables 1–3.

**Table 1:** Family Nurse Partnership outcomes and data sources

Domains	Outcomes	Years after birth	HES	NPD*
**Child outcomes (up to age 7)**				
Indicators of child maltreatment	Unplanned hospital admissions for any injury or maltreatment-related diagnosis†	0–7	✓	
	Discharge to social services at birth	0	✓	
	Child looked after (CLA)‡	4/5–7		✓
	Child in need (CiN) status‡	4/5–7		✓
	Child protection plan (CPP)‡	4/5–7		✓
Healthcare use	Unplanned hospital admissions (any diagnoses)	0–7	✓	
	A&E visits (any diagnoses)	0–7	✓	
	Referral to outpatient departments (uptake and non-attendance)	0–7	✓	
Education	School readiness measured by a good level of development in EYFSP at school entry (reception)^58^	5		✓
	Achieved expected levels at Key Stage 1 assessment (formal teacher assessment at age 7)	7		✓
	Special educational needs provision	5–7		✓
	Free school meals (eligible, applies for and receives)	5–7		✓
	Persistent absence (absent for ≥10% possible sessions)	5–7		✓
**Maternal outcomes (up to 7 years following delivery**)				
Maternal adversity	A&E attendances (any diagnoses)Unplanned hospital admissions (any diagnoses, and for violence, self-harm, or drug/alcohol abuse)^59^	0–70–7	✓✓	
Reproductive outcomes	Subsequent deliveries within 18 months of index birth	0–2	✓	
Education	Key Stage 4 assessment§ (5 A*–Cs at GCSE or equivalent)School attendance after birth¶	0–20–2		✓✓

* Including the School Census, Cin NeediN Census and CLooked AfterLA dDatabases.;

† See online supplemental table 2.Appendix Table 2;

‡ As the unique identifier for linking education and social care data is usually assigned at school entry, social care data for children only involved with social care prior to school entry cannot be linked. Therefore, we only examined CiN, CPP and CLA after school starting age (4/5 years). Thresholds for CiN status vary across the country: only assessments that have been ‘accepted’ are recorded within the data. The CiN data excludes some disabled children (those who are not receiving services from Local Authorities), and children who are receiving support from Local Authorities through early help services. We did not have the primary need code in our data and some children referred to social care services will be referred for reasons other than child maltreatment (eg, child disability).

§ Among mothers who were aged <16 years at the start of the academic year in which they reached 20 weeks of pregnancy: GCSEs areis a formal academic qualifications assessed at age 16 years.;

¶ Among mothers who were aged <15 years at the start of the academic year in which they reached 20 weeks of pregnancy.

A&EAccident and EmergencyEYFSPEarly Years Foundation Stage ProfileGCSEGeneral Certificate of Secondary EducationHES, Hospital Episode Statistics; NPD, National Pupil Database

### Statistical analysis

We first described the outcomes of interest according to maternal risk factors and enrolment in the FNP. We then compared outcomes for mothers ever enrolled in FNP and their children, versus those never enrolled, using propensity score matching based on detailed information on maternal characteristics prior to 28 weeks of gestation (table 2 and online supplemental table 4). This approach assumes that in a set of individuals who have the same propensity score, the distribution of measured baseline covariates is similar between ‘treated’ and ‘untreated’ groups, that is, between mothers who did and did not enrol in FNP.^38^

**Table 2:** Characteristics of mothers aged 13–19 years ever enrolled or not in the FNP (prior to matching)

	All mothers		Mothers enrolled in FNP		Mothers never enrolled in FNP	
	N	%	N	%	N	%
**Total**	**130 415**	**100**	**31 260**	**100**	**99 150**	**100**
Maternal age at delivery (years)						
13–15	2685	2.1	1450	4.6	1235	1.2
16–17	26 065	20.0	10 370	33.2	15 690	15.8
18–19	72 465	55.6	15 805	50.6	56 660	57.1
20*	29 205	22.4	3635	11.6	25 565	25.8
Ethnicity						
White	109 820	84.2	26 330	84.2	83 485	84.2
South Asian	3695	2.8	670	2.1	3030	3.1
Black	4650	3.6	1470	4.7	3180	3.2
Mixed/other	6840	5.2	1685	5.4	5155	5.2
Unknown	5410	4.1	1110	3.5	4300	4.3
Area-level deprivation (quintile of IMD)						
Least deprived	6810	5.2	1445	4.6	5360	5.4
2	10 410	8.0	2305	7.4	8105	8.2
3	17 855	13.7	4115	13.2	13 735	13.9
4	32 550	25	7890	25.2	24 660	24.9
Most deprived	62 630	48	15 340	49.1	47 290	47.7
Unknown	160	0.1	–	–	–	–
History of admissions/attendances with diagnoses within 2 years prior to 20 weeks of pregnancy						
Adversity (violence, self harm, substance misuse)	5475	4.2	2295	7.3	3185	3.2
Mental health (excluding self-harm/substance misuse)	3340	2.6	1400	4.5	1935	2.0
Repeat A&E attendances (≥4)	21 105	16.2	6860	21.9	14 245	14.4
Total linked to NPD (social care and education risk factors before 20 weeks of pregnancy available)	109 360	83.9	28 145	90.0	81 210	81.9
Ever excluded, in pupil referral unit or alternative provision	32 945	25.3	10 560	33.8	22 390	22.6
Ever recorded as persistently absent in a term	40 600	31.1	15 090	48.3	25 510	25.7
Ever in care	6955	5.3	3235	10.3	3720	3.8
Ever had recorded child protection plan	3885	3.0	1990	6.4	1895	1.9
Educational attainment (GCSE)†	100 270	76.9	23 785	76.1	76 485	77.1
Achieved 5 A*–C GCSEs including English/Maths	19 920	18.4	3975	14.2	15 945	19.8
Total linked to Key Stage 2 data	104 375	80.0	27 010	86.4	77 360	78.0
Achieved expected level at Key Stage 2 (Maths)	56 930	43.7	14 175	45.3	42 755	43.1
Total linked to NPD Census (FSM, SEN available)	108 365	83.1	27 995	89.6	80 365	81.1
Ever recorded as having SEN provision	56 475	43.3	17 150	54.9	39 325	39.7
Ever recorded as having FSM	61 315	47.0	18 525	59.3	42 795	43.2

Note: nNumbers have been rounded to the nearest 5 and cell sizes <10 have been supressed, in accordance with NHS Digital’s and DfE’s statistical disclosure rules for subnational analyses.

* Only including mothers aged 19 years at last menstrual period;.

† Among mothers who were aged ≥16 years at the start of the academic year in which they reached 20 weeks of pregnancy.

A&EAccident and EmergencyDfEDepartment for EducationFNPFamily Nurse PartnershipFSMfree school mealsIMDIndex of Multiple DeprivationNHSNational Health ServiceNPDNational Pupil DatabaseSENspecial educational needs

To derive propensity scores, we constructed probit regression models with FNP participation as the outcome.^38^ This provided a score reflecting the propensity for each mother in our cohort to have been enrolled in the FNP. Since we know that drivers of enrolment in the FNP vary by area, we used a multilevel structure to allow for clustering of mothers (level 1) within sites (level 2), allowing intercepts to vary for each site.^39^ We included as predictors all available maternal characteristics associated with enrolment up to 28 weeks’ gestation (at which point the vast majority of mothers have been enrolled; online supplemental table 4). For the propensity score development, model selection was informed by which predictors provided the greatest balance between FNP and non-FNP mothers in our matched cohort. We explored interactions with maternal age and by year of delivery, as we hypothesised that predictors of enrolment might vary according to these characteristics. We considered using a missingness pattern information approach to handle missing data on maternal predictors of enrolment within the propensity score model (eg, ethnicity and educational/social care predictors for the mothers who could not be linked to NPD).^40^ However, we found that explicitly modelling the missing data categories (ie, ‘unknown’ ethnicity and ‘not linked to NPD’) provided greater balance between groups.

We matched mothers enrolled in the FNP to mothers who were not enrolled, but who gave birth within the same FNP site area, on the basis of similar propensity scores (one-to-one matching without replacement, calliper width=0.01). We checked the balance between groups using standardised differences (effect sizes of 0.2, 0.5 and 0.8 are considered to be small, medium and large effect sizes, respectively).^41^ Where mothers had given birth to multiple babies, we randomly selected one child per mother to analyse: this allowed us to keep balanced numbers in each group.

The effect of FNP was estimated by evaluating outcomes for mothers who received the intervention (ie, who were enrolled in FNP) compared with the outcomes the same mothers would have experienced had they not received the intervention (in causal language, the average effect of the treatment on the treated). This effect was estimated as the difference in outcomes between matched groups. To estimate this difference, we calculated relative risks (RRs) with 95% CIs, based on generalised linear models. We used a doubly robust approach, meaning that we additionally adjusted for maternal risk factors (online supplemental table 4) within the propensity matched cohort. Model selection was based on Akaike information criterion. RRs presented are therefore adjusted RRs. All statistical analyses were conducted in Stata V.17.

#### Subgroup analyses

Interactions were used to investigate effect modification for selected outcomes according to maternal age, area-level deprivation, ethnicity, maternal history of adversity and mental health conditions, and maternal history of social care, based on previous evidence suggesting the youngest and most disadvantaged mothers are most likely to benefit from the FNP. We also explored interactions by year of delivery and region. We then presented RRs for each stratum of maternal exposure. Outcomes selected for evaluation were those with sufficient numbers to be analysed in subgroups: child unplanned admissions for maltreatment or injury up to age 2 years, a good level of development at age 5 years (school readiness), maternal unplanned admissions for any diagnosis in the 2 years following birth and subsequent births within 18 months. We did not attempt to create one high-risk group of mothers due to diminishing numbers (for example, only 85 mothers aged 13–15 years in our cohort were living in the most deprived areas).

### Changes from protocol

We were unable to evaluate mortality in this study due to large discrepancies between recording of deaths in the different data sources. We included some additional outcomes (FSM and child protection plans in the child) that were not described in the original protocol.^36^ After discussions with NFP professionals, we also evaluated duration of stay and the number of overnight and short stay (<1 day) admissions.

### Patient and public involvement

In the process of designing our study, we engaged with two groups of mothers (some who had participated in FNP, some who had not). We discussed the use of administrative data for research, linkage of health and education data without explicit consent, and the use of these data specifically for evaluating the FNP. Participants strongly agreed with sharing their data so that services could be improved and future mothers could benefit, and wanted to know how their data had been used to benefit others. Mothers were strongly supportive of taking into account maternal education and area, and wider family support for the FNP, in order to understand whether the programme worked.^42^ Our Study Steering Committee included a former adolescent mother, who was consulted throughout the study period.

## Results

### Study population

Our study cohort included 130 415 mothers. Of these, 31 260 (24%) were enrolled in FNP and 99 150 (76%) were never enrolled in FNP (table 2). Linkage to the NPD was successful for 109 635 (84.1%) of mothers. There were 110 555 mothers with 2 years of follow-up and 27 250 with 7 years of follow-up.

Mothers enrolled in FNP were younger, and more likely to be admitted to hospital for adversity-related diagnoses or to attend A&E in the 2 years prior to 20 weeks of pregnancy (table 2). FNP mothers were also more likely to have been in care or have a child protection plan, more likely to be recorded as having SEN provision, FSM and be in the most deprived quintile according to Income Deprivation Affecting Children Index, more likely to have been excluded or be persistently absent, and less likely to achieve 5 A*–Cs at General Certificate of Secondary Education.

Unadjusted comparisons of outcomes according to enrolment in FNP are presented in online supplemental tables 5–9.

### Propensity score-matched cohort

FNP mothers tended to have higher propensity scores (median=0.39) than non-FNP mothers (median=0.31), meaning that these mothers had more risk factors for enrolment than those not enrolled. However, there was a good overlap of propensity scores between groups (online supplemental figure 2): we were able to include 94.9% of mothers in the matched analysis for births between April 2010 and 2019 and 95.7% of mothers in the cohort with 2 years of follow-up, and 99.9% of mothers in the cohort with 7 years of follow-up. There were no large imbalances between matched groups (all standardised differences <0.1; online supplemental figure 3).

#### Indicators of child maltreatment

**Figure 1:**
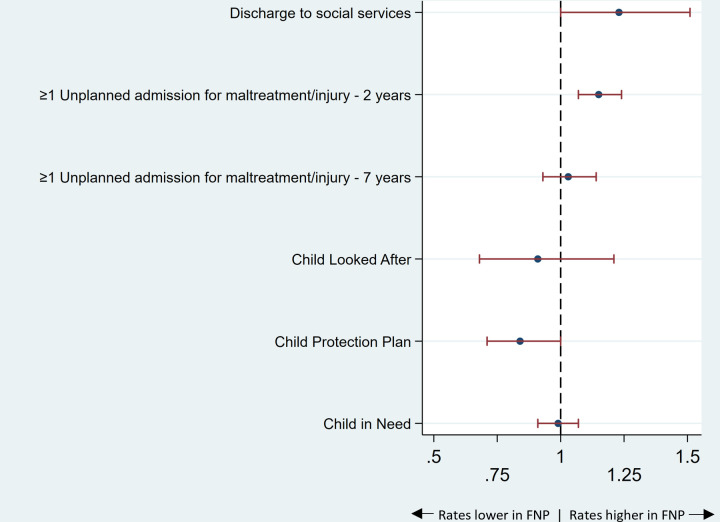
Indicators of child maltreatment: adjusted relative risks* and 95% CIs comparing outcomes for mothers enrolled in the FNP versus mothers who were not enrolled, for mothers aged 13–19 years and giving birth in an area in which FNP was offered at the time of pregnancy, in the propensity-matched cohort. *Relative risks are adjusted for all maternal characteristics prior to enrolment in table 2. FNP, Family Nurse Partnership.

There was an increased risk of unplanned admissions for maltreatment/injury-related diagnoses in the 2 years following birth among children of mothers who were enrolled in the FNP compared with those who were not, in the matched cohort (figure 1 and online supplemental table 10). There was weak evidence that FNP was associated with an increased risk of a hospital record indicating discharge to social services at birth (RR 1.23; 95% CI 1.00, 1.51), and a decreased risk of a child protection plan up to 7 years after birth (RR 0.84; 95% CI 0.71, 1.00). The median length of stay for children admitted to hospital for maltreatment/injury-related diagnoses was the same in both groups (1 day; IQR 0.5–1, where 0.5 days indicates an admission and discharge on the same day; online supplemental table 12).

#### Child health, developmental and educational outcomes

**Figure 2:**
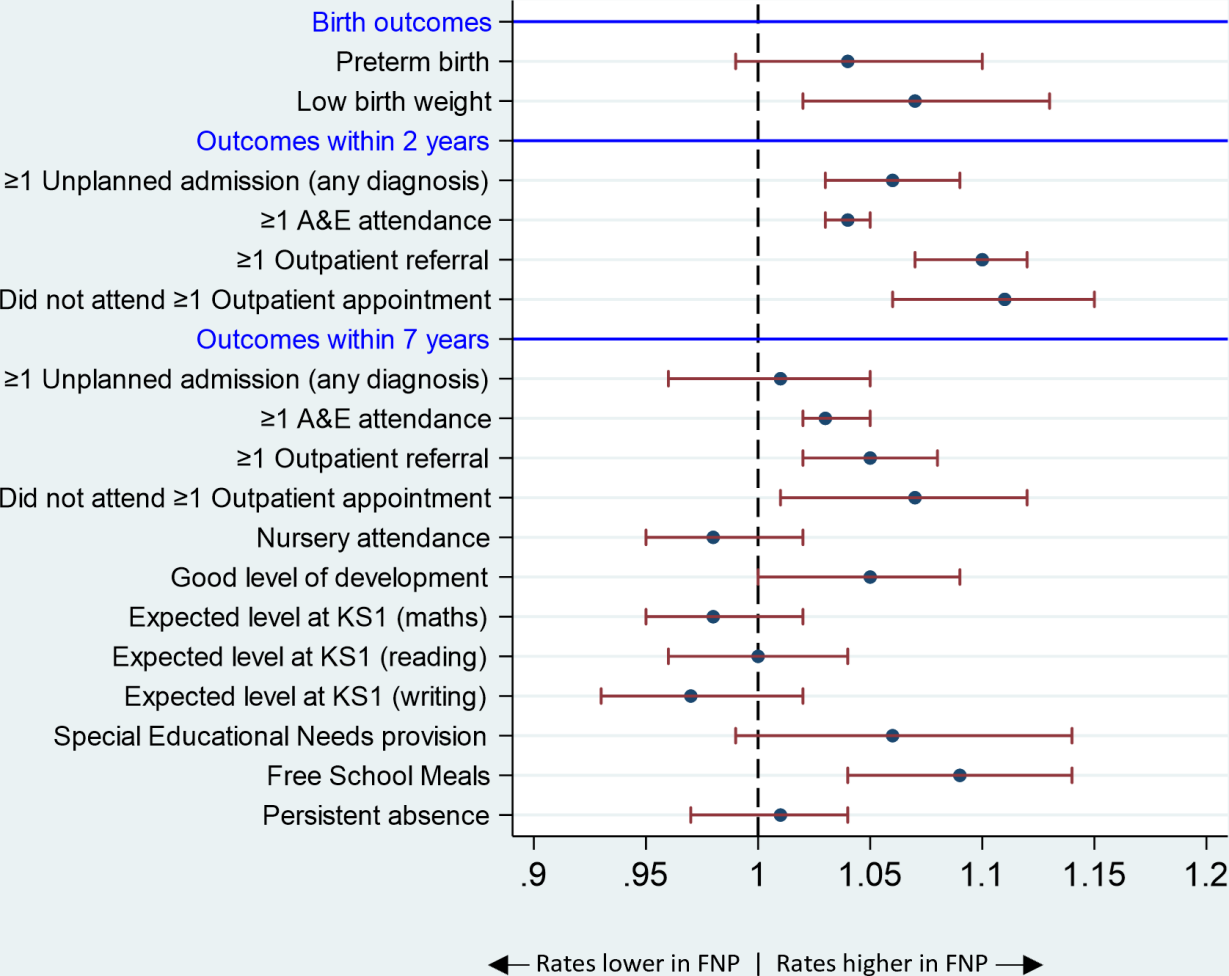
Child health, developmental and educational outcomes: adjusted relative risks* and 95% CIs comparing mothers enrolled in the FNP versus mothers who were not enrolled, for mothers aged 13–19 years and giving birth in an area in which FNP was offered at the time of pregnancy, in the propensity-matched cohort. *Relative risks are adjusted for all maternal characteristics prior to enrolment in table 2. A&E, Accident and Emergency; FNP, Family Nurse Partnership; KS, Key Stage.

There was an increased risk of low birth weight among mothers who were enrolled in the FNP versus those who were not (figure 2 and online supplemental table 11). There was also an increased risk of unplanned admission for any diagnoses (in the 2 years following birth) and for A&E attendances (in the 2 and 7 years following birth).

There was weak evidence that children born to FNP mothers were more likely to achieve a good level of development (school readiness) at age 5 years (RR 1.05; 95% CI 1.00, 1.09) than those born to mothers who were not enrolled (online supplemental table 12). Children in the FNP arm were also more likely to be recorded as having FSM.

#### Maternal outcomes

**Figure 3:**
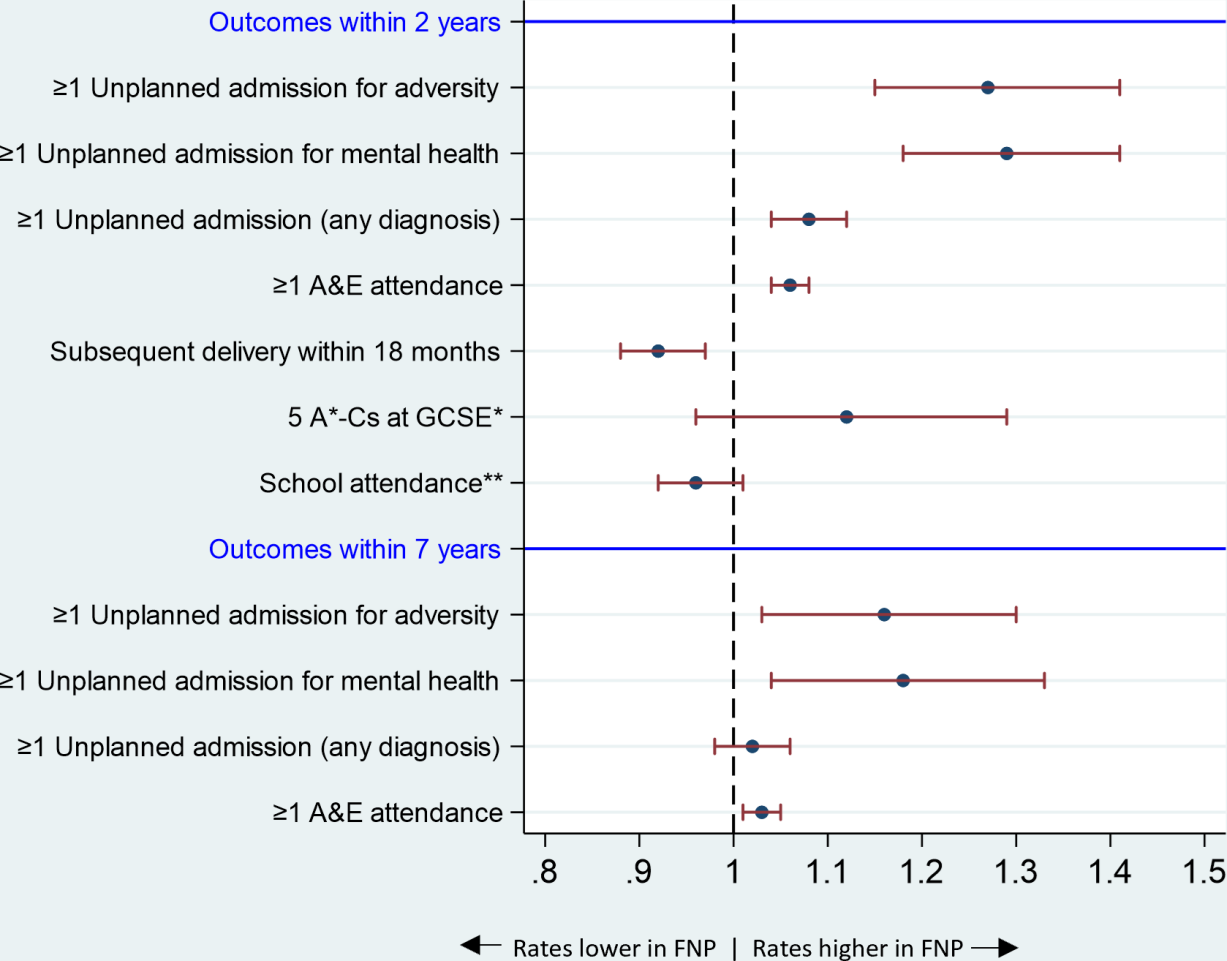
Maternal outcomes: adjusted relative risks∧ and 95% CIs comparing outcomes for mothers enrolled in the FNP versus mothers who were not enrolled, for mothers aged 13–19 years and giving birth in an area in which FNP was offered at the time of pregnancy, in the propensity-matched cohort. ∧Relative risks are adjusted for all maternal characteristics prior to enrolment in table 2. *Among mothers who were aged <16 years at the start of the academic year in which they reached 20 weeks’ gestation. **Among mothers who were aged <15 years at the start of the academic year in which they reached 20 weeks’ gestation. A&E, Accident and Emergency; FNP, Family Nurse Partnership; GCSE, General Certificate of Secondary Education.

Mothers who enrolled in the FNP were more likely to have unplanned admissions for adversity-related diagnoses, mental health conditions or any diagnoses, and A&E attendances in the 2 and 7 years following birth, compared with those who were not enrolled (figure 3 and online supplemental table 14). However, these mothers were less likely to have a repeat birth within 18 months of the index birth (RR 0.92; 95% CI 0.88, 0.97).

#### Subgroup analyses

There were no statistically significant interactions between enrolment in FNP and maternal age, ethnicity, area-level deprivation, maternal contact with social care services, child sex or year of delivery (online supplemental figures 4–7).

## Discussion

Our population-based study of the FNP in England supports previous evidence showing no benefit of the FNP on child maltreatment outcomes, and adds to the broader literature on the effectiveness of home visiting programmes for reducing maltreatment. We observed a small increase in the number of children achieving a good level of development (school readiness) at age 5 years associated with enrolment in the FNP, supporting similar findings from the Building Blocks 2:6 trial, and fewer maternally reported developmental concerns at age 2 years in the FNP arm of the original Building Blocks trial.^21 23^ We also observed a reduction in the number of subsequent deliveries within 18 months for mothers enrolled in FNP. Birth spacing of more than 18 months is key for promoting maternal education and engagement in the workforce, and preventing harm to the mother and child, and we show that it may be possible for the programme to influence birth spacing and therefore the maternal life course.^43 44^ However, this result should be interpreted cautiously as we could not measure abortions or miscarriages, and we do not know how many mothers in each group became pregnant but chose, or were unable, to continue their pregnancy.

A major strength of our study was the large sample size resulting from the use of a population-based cohort of all first-time adolescent mothers giving birth in English NHS hospitals between 2010 and 2019. Linking 99% of FNP mothers to HES gave us a highly generalisable cohort of FNP mothers. Another strength was our use of objective measures of indicators of maltreatment and child development. We also spent a significant amount of time talking to FNP teams, including observing a home visit, in order to understand the perspectives of family nurses and the mothers and families they represent. This enhanced our ability to interpret the results we observed in the data.

There are a number of challenges inherent to evaluations using administrative data, and findings in this report should be interpreted in the context of three main limitations.^45^

First, although we carefully designed and assessed the propensity score analysis strategy, our approach is subject to the assumption that conditional on the propensity score, the distribution of characteristics between groups was balanced. However, there may be residual confounding as we could only control for the fairly crude maternal risk factors associated with enrolment in FNP that are captured in administrative data. We know that mothers enrolled in the FNP had more risk factors for adverse outcomes than those who were not enrolled (table 2). Although propensity score matching has a number of limitations, it can help most where there are high levels of imbalance as in this setting.^46^ However, even after matching, the increased rates of low birthweight babies, babies discharged to social services and of maternal unplanned admissions post-pregnancy for adversity and mental health-related diagnoses that we observed in the FNP group indicate that there may be residual confounding and bias by indication. Additional information on other important characteristics including less severe mental health conditions, family support and risk of unstable housing would have improved our ability to overcome this confounding. Given we know that mothers enrolled in FNP had more indicators of vulnerability at enrolment than the comparison group, such residual confounding could have limited our ability to detect beneficial effects of the programme. The weak evidence for small improvements in school readiness as measured by a good level of development in the EYFSP and reductions in the number of rapid repeat pregnancies may therefore reflect larger positive effects of the programme. However, these outcomes should still be considered in the context of being the only ‘positive’ effects among the many outcomes that were evaluated and the statistical power provided by such a large sample size. These challenges are relevant to other studies aiming to use administrative data to evaluate public health interventions.^45^

Second, outcomes captured in administrative data can be difficult to interpret. For example, the increased rates of unplanned admissions and A&E attendances in the mother and child associated with FNP may be viewed as contradictory to the aims of improving child health, but may actually reflect appropriate care seeking as a result of advice and support from family nurses. The increase in unplanned admissions in the FNP group was mostly driven by short stay admissions (online supplemental table 12), indicating that mothers could have been seeking help for relatively minor conditions. In addition, babies born to mothers enrolled in the FNP were more likely to be born preterm and with low birth weight: these are health conditions which are influenced by pre-conception health, and babies with these conditions would be expected to have appropriately increased rates of hospital contacts throughout childhood. Increased rates of admissions in the mother could also be interpreted as demonstrating that family nurses can have a long-term effect on maternal healthcare-seeking behaviours. Family nurses work with mothers to explore the trauma that many of them have experienced in the past and to become more insightful about their own needs. Cuts to mental health services have made making appropriate referrals difficult, which may lead to mothers presenting in A&E. However, we could only measure the most severe problems, as we only identified cases that resulted in a hospital admission record. We did not have data on primary care, which could have provided more information on outcomes that were not severe enough to result in a hospital admission.

Our finding of weak evidence of a reduction in the number of child protection plans associated with FNP (and similar directions of non-significant associations for child in need (CiN) and child looked after (CLA)) reinforces the complexity of understanding the mechanisms underlying these outcomes. Others have argued that given the ethical, clinical and legal mandate that family nurses have to ensure that children are protected through linkages with appropriate services, increased rates of social care contacts might well be expected in mothers enrolled in the FNP, reflecting earlier and more comprehensive surveillance.^47 48^ Family nurses are in a unique position to identify early problems and to start processes for safeguarding, which may explain the increased rates of discharge to social services at birth. However, we measured CiN and CLA in school-age children, who would not have still been seen by the family nurse and who would therefore be less likely to have been affected by surveillance bias.

Third, FNP might have positive effects on a range of other outcomes that are not captured in administrative data. This study was not able to identify effects on changes in self-reported parental mental health, sense of self, well-being, confidence, behaviour and parent–child engagement and interaction, including for fathers. Nuances in behaviour change are difficult to measure: for example, a mother may not give up smoking completely, but may change how she smokes, by not smoking in the home and not allowing others to smoke in the home. Further work is also needed to understand how differing dose or intensity of the programme might be related to maternal characteristics and to outcomes.

Existing qualitative work demonstrates overwhelming support for the programme from mothers who have been enrolled, and from family nurses who can see the changes and impact that the intensive service has provided for the families they have worked with.^49 50^ However, there is a lack of conclusive systematic evidence supporting interventions for preventing child maltreatment more generally.^1651^,^53^ Expecting to detect effects of home visiting that starts in pregnancy on birth outcomes and on relatively insensitive child development measures may also be unreasonable in the context of the social disadvantage, discrimination and other challenges that adolescent mothers face before, during and after pregnancy.^54^ Indeed, a recent trial of 5670 Medicaid-eligible nulliparous pregnant mothers recruited between 2016 and 2020 in South Carolina found no evidence of an effect on birth outcomes (preterm birth, low birth weight, small for gestational age and perinatal death).^55^ Strategies to address the root causes of social disadvantage experienced by young mothers are therefore also needed.

Despite reductions in adolescent pregnancies over recent decades, there remains a significant population of young and vulnerable mothers in England who need intensive support. Currently, the majority of these mothers are not receiving support from FNP, as it is not offered in all areas, and only offered to around one in four mothers in areas in which it is commissioned.^26^ There is strong support for FNP locally, and FNP practitioners report that mothers participating in the programme develop more reflective parenting and awareness of their child’s needs. Without better evidence, removing support for young mothers could be harmful, especially in the context of increasing social disadvantage and widespread health visitor shortages that are already putting pressure on other services.^56^ More research is needed to understand which elements of intensive interventions are most effective, for whom and when, and whether it is better to commission highly intensive services for a small portion of the target population, or to extend and enhance universal services to better support all adolescent mothers.^18 57^

## supplementary material

10.1136/bmjph-2023-000514 online supplemental file 1

## Data Availability

Data may be obtained from a third party and are not publicly available.

## References

[1] ONS (2022). Birth characteristics 2019. office for National Statistics. https://www.ons.gov.uk/peoplepopulationandcommunity/birthsdeathsandmarriages/livebirths/datasets/birthcharacteristicsinenglandandwales.

[2] Crawford C, Cribb J, Kelly E (2019). Teenage pregnancy in England 2013. https://ifs.org.uk/publications/teenage-pregnancy-england.

[3] Wellings K, Palmer MJ, Geary RS (2016). Changes in conceptions in women younger than 18 years and the circumstances of young mothers in England in 2000–12: an observational study. Lancet.

[4] Bellis MA, Hughes K, Leckenby N (2014). National household survey of adverse childhood experiences and their relationship with resilience to health-harming behaviors in England. BMC Med.

[5] Rigsby DC, Macones GA, Driscoll DA (1998). Risk factors for rapid repeat pregnancy among adolescent mothers: A review of the literature. J Pediatr Adolesc Gynecol.

[6] Restrepo-Méndez MC, Lawlor DA, Horta BL (2015). The Association of maternal age with birthweight and gestational age: A cross-cohort comparison. Paediatr Perinat Epidemiol.

[7] Cunnington AJ (2001). What’s so bad about teenage pregnancy. J Fam Plann Reprod Health Care.

[8] Stier DM, Leventhal JM, Berg AT (1993). Are children born to young mothers at increased risk of Maltreatment. Pediatrics.

[9] Gonzalez A, MacMillan HL (2008). Preventing child Maltreatment: an evidence-based update. J Postgrad Med.

[10] Jutte DP, Roos NP, Brownell MD (2010). The ripples of adolescent motherhood: social, educational, and medical outcomes for children of teen and prior teen mothers. Acad Pediatr.

[11] Wiggins M, Oakley A, Sawtell M (2019). Teenage Parenthood and social exclusion: a multi-method study - summary report of findings. https://discovery.ucl.ac.uk/id/eprint/10003007/1/Wiggins2005TeenageParenthood.pdf.

[12] Lawlor D, Shaw M, Johns S (2001). Teenage pregnancy is not a public health problem. BMJ.

[13] Sweet MA, Appelbaum MI (2004). Is home visiting an effective strategy? A meta-analytic review of home visiting programs for families with young children. Child Dev.

[14] Kim H, Song EJ, Windsor L (2024). Evidence-based home visiting provisions and child Maltreatment report rates: County-level analysis of US National data from 2016 to 2018. Child Maltreat.

[15] Avellar SA, Supplee LH (2013). Effectiveness of home visiting in improving child health and reducing child Maltreatment. Pediatrics.

[16] Euser S, Alink LR, Stoltenborgh M (2015). A gloomy picture: a meta-analysis of randomized controlled trials reveals disappointing effectiveness of programs aiming at preventing child Maltreatment. BMC Public Health.

[17] Casillas KL, Fauchier A, Derkash BT (2016). Implementation of evidence-based home visiting programs aimed at reducing child Maltreatment: A meta-analytic review. Child Abuse Negl.

[18] Gubbels J, van der Put CE, Stams G-JJM (2021). Components associated with the effect of home visiting programs on child Maltreatment: A meta-analytic review. Child Abuse Negl.

[19] Macmillan HL, Wathen CN, Barlow J (2009). Interventions to prevent child Maltreatment and associated impairment. Lancet.

[20] Olds DL, Hill PL, O’Brien R (2003). Taking preventive intervention to scale: the nurse-family partnership. Cognitive and Behavioral Practice.

[21] Robling M, Lugg-Widger F, Cannings-John R (2021). The family nurse partnership to reduce Maltreatment and improve child health and development in young children:the BB:2–6 routine data-linkage follow-up to earlier RCT. *Public Health Res*.

[22] Dartington Service Design Lab Family nurse partnership national unit. FNP ADAPT: using evidence, pragmatism and collaboration to change the FNP programme in England 2020. https://www.fnp.nhs.uk/media/1359/fnp_adapt_report_web.pdf.

[23] Robling M, Bekkers M-J, Bell K (2016). Effectiveness of a nurse-led intensive home-visitation programme for first-time teenage mothers (building blocks): a pragmatic randomised controlled trial. Lancet.

[24] Olds D (2016). Building evidence to improve maternal and child health. Lancet.

[25] Barlow J, Barnes J, Sylva K (2016). Questioning the outcome of the building blocks trial. Lancet.

[26] Cavallaro FL, Gilbert R, Wijlaars LP (2022). Characteristics of Enrolment in an intensive home-visiting programme among eligible first-time adolescent mothers in England: a linked administrative data cohort study. J Epidemiol Community Health.

[27] Robling M, Cannings-John R, Channon S (2018). What is usual care for teenagers expecting their first child in England? A process evaluation using key informant mapping and participant survey as part of the building blocks randomised controlled trial of specialist home visiting. BMJ Open.

[28] Booth CM, Tannock IF (2014). Randomised controlled trials and population-based observational research: partners in the evolution of medical evidence. Br J Cancer.

[29] Bryar RM, Cowley DSA, Adams CM (2017). Health visiting in primary care in England: a crisis waiting to happen. Br J Gen Pract.

[30] Kennedy-Martin T, Curtis S, Faries D (2015). A literature review on the Representativeness of randomized controlled trial samples and implications for the external validity of trial results. Trials.

[31] Herbert A, Wijlaars L, Zylbersztejn A (2017). Data resource profile: hospital episode Statistics admitted patient care (HES APC). Int J Epidemiol.

[32] Mc Grath-Lone L, Harron K, Dearden L (2016). Data resource profile: children looked after return (CLA). Int J Epidemiol.

[33] Harron K, Gilbert R, Cromwell D (2016). Linking data for mothers and babies in de-identified electronic health data. PLoS One.

[34] Harron KL, Doidge JC, Knight HE (2017). A guide to evaluating linkage quality for the analysis of linked data. Int J Epidemiol.

[35] Libuy N, Harron K, Gilbert R (2021). Linking education and hospital data in England: linkage process and quality. *IJPDS*.

[36] Cavallaro FL, Gilbert R, Wijlaars L (2020). Evaluating the real-world implementation of the family nurse partnership in England: protocol for a data linkage study. BMJ Open.

[37] Cannings-John R, Lugg-Widger F, Lau M (2020). Evaluation of family nurse partnership: methods and process of evaluation - Appendix 1: logic model. https://www.gov.scot/publications/evaluation-family-nurse-partnership-scotland-methods-paper-process-success-linkages/pages/13/.

[38] Austin PC (2011). An introduction to propensity score methods for reducing the effects of confounding in observational studies. Multivariate Behav Res.

[39] Li F, Zaslavsky AM, Landrum MB (2013). Propensity score weighting with Multilevel data. Stat Med.

[40] Blake HA, Leyrat C, Mansfield KE (2020). Propensity scores using Missingness pattern information: a practical guide. Stat Med.

[41] Austin PC (2009). Balance diagnostics for comparing the distribution of baseline covariates between treatment groups in propensity-score matched samples. Stat Med.

[42] Harron K (2022). It did mean a lot": what public engagement with teenage mothers taught us about our research. https://blogs.ucl.ac.uk/public-engagement/2019/05/07/engaging-with-teenage-mothers/.

[43] Conde-Agudelo A, Rosas-Bermudez A, Castaño F (2012). Effects of birth spacing on maternal, perinatal, infant, and child health: a systematic review of causal mechanisms. Stud Fam Plann.

[44] Conde-Agudelo A, Rosas-Bermúdez A, Kafury-Goeta AC (2006). Birth spacing and risk of adverse perinatal outcomes: a meta-analysis. JAMA.

[45] Cavallaro F, Cannings-John R, Lugg-Widger F Lessons learned from using linked administrative data to evaluate the family nurse partnership in England and Scotland. *IJPDS*.

[46] King G, Nielsen R (2019). Why propensity scores should not be used for matching. Polit Anal.

[47] Olds D, Henderson CR, Kitzman H (1995). Effects of Prenatal and infancy nurse home visitation on surveillance of child Maltreatment. Pediatrics.

[48] Olds D (2022). Improving the report of the building blocks 2-6 study. BMJ Open.

[49] Robling M (2015). The Building Blocks Trial. Evaluating the Family Nurse Partnership Programme in England: A Randomised Controlled Trial.

[50] Owens R (2020). Digging down and scaling up: a psychosocial exploration of the Family Nurse Partnership.

[51] Mikton C, Butchart A (2009). Child Maltreatment prevention: a systematic review of reviews. Bull World Health Organ.

[52] Vlahovicova K, Melendez-Torres GJ, Leijten P (2017). Parenting programs for the prevention of child physical abuse recurrence: A systematic review and meta-analysis. Clin Child Fam Psychol Rev.

[53] Chen M, Chan KL (2016). Effects of parenting programs on child Maltreatment prevention:A meta-analysis. Trauma Violence Abuse.

[54] Moniz MH, Low LK, Stout MJ (2022). Intensive nurse home visiting program and adverse birth outcomes. JAMA.

[55] McConnell MA, Rokicki S, Ayers S (2022). Effect of an intensive nurse home visiting program on adverse birth outcomes in a Medicaid-eligible population: A randomized clinical trial. JAMA.

[56] Wilkinson E (2022). Health visitor shortages are risking child health and piling pressure on other services. BMJ.

[57] Goodman WB, Dodge KA, Bai Y (2019). Randomized controlled trial of family connects: effects on child emergency medical care from birth to 24 months. Dev Psychopathol.

[58] Department for Education, National Statistics (2019). Early years foundation stage profile results in England, 2018.

[59] Herbert A, Gilbert R, González-Izquierdo A (2015). Violence, self-harm and drug or alcohol misuse in adolescents admitted to hospitals in England for injury: a retrospective cohort study. BMJ Open.

