# Supplementary material for: Intensive home visiting for adolescent mothers in the Family Nurse Partnership in England 2010–2019: a population-based data linkage cohort study using propensity score matching
Source: BMJ Public Health. 2024 Feb 20;2(1):e000514. doi: 10.1136/bmjph-2023-000514 (PMC11812803; doi:10.1136/bmjph-2023-000514)

Appendices

Appendix Figure 1: Time periods and ages of cohort members included in the study. Shaded boxes indicate age of member during study period (blue); look-back period for health data (grey); follow-up (light blue); and age of child during follow up (green). The look back period is the earliest of age 11 (for Key Stage 2 results), or 5 years prior to delivery (in HES).

	Data source	Year of delivery of index child																		
		2001	2002	2003	2004	2005	2006	2007	2008	2009	2010	2011	2012	2013	2014	2015	2016	2017	2018	2019
	Births																			
	FNP																			
	HES																			
	NPD																			
	CLA																			
	CiN																			
Year of birth of mother	1991	10	11	12	13	14	15	16	17	18	19	20	21	22	23	24	25	26		
	1992	9	10	11	12	13	14	15	16	17	18	19	20	21	22	23	24	25	26	
	1993	8	9	10	11	12	13	14	15	16	17	18	19	20	21	22	23	24	25	26
	1994	7	8	9	10	11	12	13	14	15	16	17	18	19	20	21	22	23	24	25
	1995	6	7	8	9	10	11	12	13	14	15	16	17	18	19	20	21	22	23	24
	1996	5	6	7	8	9	10	11	12	13	14	15	16	17	18	19	20	21	22	23
	1997	4	5	6	7	8	9	10	11	12	13	14	15	16	17	18	19	20	21	22
	1998	3	4	5	6	7	8	9	10	11	12	13	14	15	16	17	18	19	20	21
	1999	2	3	4	5	6	7	8	9	10	11	12	13	14	15	16	17	18	19	20
	2000	1	2	3	4	5	6	7	8	9	10	11	12	13	14	15	16	17	18	19
	2001	0	1	2	3	4	5	6	7	8	9	10	11	12	13	14	15	16	17	18
	2002		0	1	2	3	4	5	6	7	8	9	10	11	12	13	14	15	16	17
	2003			0	1	2	3	4	5	6	7	8	9	10	11	12	13	14	15	16
	2004				0	1	2	3	4	5	6	7	8	9	10	11	12	13	14	15
	2005					0	1	2	3	4	5	6	7	8	9	10	11	12	13	14
	2006						0	1	2	3	4	5	6	7	8	9	10	11	12	13
	-	-	-	-	-	-	-	-	-	-	-	-	-	-	-	-	-	-	-	-
Year of birth of child	2010										0	1	2	3	4	5	6	7		
	2011											0	1	2	3	4	5	6	7	
	2012												0	1	2	3	4	5	6	7
	2013													0	1	2	3	4	5	6
	2014														0	1	2	3	4	5
	2015															0	1	2	3	4
	2016																0	1	2	3
	2017																	0	1	2

FNP: Family Nurse Partnership; HES: Hospital Episode Statistics; NPD: National Pupil Database; CLA: Child Looked After; CiN: Child in Need

Appendix Table 1: Description of outcomes included in the study

Outcome	Description
**Indicators of child maltreatment**	- Discharge from hospital to social services at birth<br>- Unplanned hospital admission for injury or maltreatment-related diagnoses (up to age 2 or 7, see Appendix Table 2 for ICD-10 code lists)<br>- Records of being a Child in Need (CiN), as having a Child Protection Plan (CPP), or being a Child Looked After (CLA).
**Child health, developmental and educational outcomes**	- Preterm birth (<37 weeks of pregnancy) and low birth weight (<2500g)<br>- A&E attendance and unplanned hospital admissions for any diagnosis (up to age 2 or 7)<br>- For children reaching school age, we examined the effect of the FNP on:<br>o School readiness as measured by the percentage of each group achieving a Good Level of Development as recorded within the Early Years Foundation Stage Profile (EYFSP) at age 5. A "good" level of development is recorded if children are at the expected level for the 12 early learning goals within the 5 areas of learning relating to: communication and language; personal, social and emotional development; physical development; literacy; and mathematics.<br>o Persistent absence (absent for ≥10% of possible school sessions)<br>o Achieving expected levels at Key Stage 1 (age 7) for Mathematics, Reading and Writing<br>o Recording of Special Educational Needs provision<br>o Recording of Free School Meal eligibility (pupils are recorded as eligible if a claim for free school meals has been made by them or on their behalf by parents)<br>o Nursery attendance as recorded in the Early Years Census.
**Maternal outcomes**	- Unplanned hospital admissions for adversity-related reasons (violence, self-harm, and drug/alcohol abuse), or for mental health-related diagnoses, after delivery (Appendix Table 3)<br>- Subsequent live births within 18 months of first live birth as recorded in HES.<br>- Amongst mothers who had not previously had the opportunity to take GCSEs, we evaluated the percentage in each group who achieved 5 A*-C grades including English/Maths at GCSE level (or equivalent), in the two years after delivery.<br>- Amongst mothers who would still have been of school age in the year following the academic year in which they reached 20 weeks of pregnancy, we evaluated enrolment in school up to Year 11.

Appendix Table 2: ICD-10 code lists for child maltreatment and health care utilisation-related outcomes. Unplanned hospital admissions with any of the specified ICD-10 codes were considered to be maltreatment or injury related.

Group	Description	ICD-10 Code	Age restriction
**Maltreatment and injury-related**			
**Injury & ingestion**	Injuries to the head (includes open wounds, fractures, crushing and dislocation)	S00-S09	N/A
	Injuries to the neck	S10-S19	N/A
	Injuries to the thorax	S20-S29	N/A
	Injuries to the abdomen, lower back, lumbar spine and pelvis	S30-S39	N/A
	Injuries to the shoulder and upper arm	S40-S49	N/A
	Injuries to the elbow and forearm	S50-S59	N/A
	Injuries to the wrist and hand	S60-S69	N/A
	Injuries to the hip and thigh	S70-S79	N/A
	Injuries to the knee and lower leg	S80-S89	N/A
	Injuries to the ankle and foot	S90-S99	N/A
	Injuries involving multiple body regions	T00-T07	N/A
	Injuries to unspecified part of trunk, limb or body region	T08-T14	N/A
	Effects of foreign body entering through natural orifice	T15-T19	N/A
	Burns and corrosions	T20-T32	N/A
	Frostbite	T33-T35	N/A
	Poisoning by drugs, medicaments and biological substances	T36-T50	N/A
	Toxic effects of substances chiefly non-medicinal as to source (sting, alcohol, solvents, etc.)	T51-T65	N/A
	Other and unspecified effects of external causes (effects of radiation, heat and light, hypothermia, electric shock, asphyxiation, food deprivation)	T66-T78	N/A
	Accidental poisoning by and exposure to noxious substances	X40-X49	N/A
**Maltreatment**	Maltreatment syndromes	T74	N/A
	Neglect and abandonment	Y06	N/A
	Other maltreatment	Y07	N/A
	Effects of other deprivation (hunger, thirst, exhaustion due to exposure or excessive exertion)	T73	N/A
	Assault	X85-Y05 Y08-Y09	N/A
**Maltreatment markers for infants <1yr**	Intracranial injuries	S06	<1yr
	Long-bone fractures	S42.2-S42.4, S42.7-S42.8, S52, S72, S82, T10, T12	<1yr
**Maltreatment markers for children ≤4yrs**	Traumatic brain injuries	S06, S09.7-S09.8, T90.5	≤4yrs
	Retinal haemorrhage	H35.6	≤4yrs
	Rib fractures	T29-T32	≤4yrs

Code lists were derived from the following studies:

- Gilbert R, Fluke J, O'Donnell M, et al. Child maltreatment: variation in trends and policies in six developed countries. Lancet 2011; 379 (9817):758-72
- Syed S, Ashwick R, Schlosser M, et al Predictive value of indicators for identifying child maltreatment and intimate partner violence in coded electronic health records: a systematic review and meta-analysis. Archives of Disease in Childhood 2021;106:44-53.
- Robling M, Lugg-Widger F, Cannings-John R, Sanders J, Angel L, Channon S, et al. The Family Nurse Partnership to reduce maltreatment and improve child health and development in young children: the BB:2–6 routine data-linkage follow-up to earlier RCT. Public Health Res 2021;9(2).

Appendix Table 3: ICD-10 code lists for maternal hospital admissions related to adversity, mental health, and chronic conditions. Unplanned maternal hospital admissions with any of the specified ICD-10 codes were considered to be adversity-related or mental health-related. Mothers with any hospital admissions relating to a chronic condition were considered to have a chronic condition.

Group	Description	ICD10 Code
**Adversity-related admissions**		
**Violence**	Maltreatment syndromes	T74
	Effects of other deprivation (extreme neglect)	T73
	Perpetrator of neglect and other maltreatment syndromes	Y06, Y07
	Assault by bodily force and sexual assault	Y04, Y05
	Other types of assault	X85-Y03, Y08-Y09
	Events of undetermined intent	Y20-Y34
	Examination and observation following other inflicted injury	Z04.5
	Examination and observation for other reasons: request for expert evidence	Z04.8
**Self-harm**	Sequelae of intentional self-harm	Y87.0
	Intentional self-poisoning by and exposure to … drugs	X60-X63
	…other and unspecified drugs, medicaments and biological substances	X64
	…alcohol	X65
	…organic solvents and halogenated hydrocarbons and their vapours	X66
	…other gases and vapours	X67
	…pesticides	X68
	…other and unspecified chemicals and noxious substances	X69
	Intentional self-harm by… hanging, strangulation and suffocation	X70
	…drowning and submersion	X71
	…firearm discharge	X72-X74
	…explosive material	X75
	…smoke, fire and flames, or steam, hot vapours and hot objects	X76-X77
	…sharp/blunt objects	X78-X79
	...jumping from a high place	X80
	…jumping or lying before a moving object, or crashing a motor vehicle	X81-82
	…other specified means	X83
	…unspecified means	X84
**Substance misuse**	Mental and behavioural disorders due to psychoactive substance use	F11-F16, F19
	Finding of drugs not normally found in blood	R78.1-R78.5
	Poisoning by drugs, medicaments and biological substances	T36-T50 (not T50.6)
	Poisoning, undetermined intent	Y10-Y14
	Drug rehabilitation	Z50.3
	Drug abuse counselling and surveillance	Z71.5
	Drug use	Z72.2

	Mental and behavioural disorders due to use of volatile solvents	F18
	Accidental poisoning by and exposure to noxious substances	X40–X44, X46-X49
	Poisoning by chemical or noxious substance, undetermined intent	Y16-Y19
	Special epileptic syndromes - (related to alcohol, drugs, etc.)	G40.5
	Blood-alcohol and blood-drug test	Z04.0
	Alcohol-induced pseudo-Cushing's syndrome	E24.4
	Mental and behavioural disorders due to use of alcohol	F10
	Degeneration of nervous system due to alcohol	G31.2
	Alcoholic polyneuropathy	G62.1
	Alcoholic myopathy	G72.1
	Alcoholic cardiomyopathy	I42.6
	Alcoholic gastritis	K29.2
	Alcoholic liver disease	K70
	Alcohol-induced acute pancreatitis	K85.2
	Alcohol-induced chronic pancreatitis	K86.0
	Maternal care for (suspected) damage to fetus from alcohol	O35.4
	Finding of alcohol in blood	R78.0
	Poisoning: antidotes and chelating agents, not elsewhere classified	T50.6
	Toxic effect of alcohol	T51
	Accidental poisoning by exposure to alcohol	X45
	Poisoning by exposure to alcohol, undetermined intent	Y15
	Evidence of alcohol involvement determined by blood alcohol level	Y90
	Evidence of alcohol involvement determined by level of intoxication	Y91
	Alcohol rehabilitation	Z50.2
	Alcohol abuse counselling and surveillance	Z71.4
	Alcohol use	Z72.1
**Mental health conditions / behavioural disorders (excluding those falling under adversity or chronic conditions)**		
	Organic, including symptomatic, mental disorders	F00-F09*
	Schizophrenia, schizotypal and delusional disorders	F20-F29
	Mood [affective] disorders	F30-F39
	Neurotic, stress-related and somatoform disorders	F40-F48
	Behavioural syndromes associated with physiological disturbances and physical factors	F50-F59**
	Disorders of adult personality and behaviour	F60-F69***
	Mental retardation	F70-F79
	Disorders of psychological development	F80-F89
	Behavioural and emotional disorders with onset usually occurring in childhood and adolescence	F90-F98
	Sedatives, hypnotics and antianxiety drugs	Y47
	Psychotropic drugs, not elsewhere classified	Y49
**Chronic conditions (except those falling under mental health conditions)**		
**Cancer/blood disorders**	Neoplasms	C00-C97, D00-D02, D05-D09, D12, D13, D14.1-D14.4, D15, D20, D32-D35, D37-

		D48, D63.0, E34.0, E88.3, G13.0, G13.1, G53.3, G55.0, G63.1, G73.1, G73.2, G94.1, M36.0, M36.1, M49.5, M82.0, M90.6, M90.7, N08.1, N16.1, Y43.1-Y43.3, Y84.2, Z08, Z51.0-Z51.2, Z54.1, Z54.2, Z85, Z86.0, Z92.3
	Immunological disorders	D80-D84, G53.2, Q98.0
	Anaemia and other blood disorders	D50, D56.0-D56.2, D56.4, D56.8, D56.9, D57.0-D57.2, D57.8, D58, D61.0, D61.9, D64, D66, D67, D68.0-D68.2, D68.4-D68.9, D69, D70-D76, M36.2-M36.4, M90.4, N08.2, Z86.2
**Chronic infections**	HIV	B20-B24, F02.4, R75, Z21
	Tuberculosis	A15-A19, E35.0, K23.0, K67.3, K93.0, M01.1, M49.0, P37.0
	Other	A50, A81, B18, B37.1, B37.5, B37.6, B37.7, B38.1, B39.1, B40.1, B44.0, B44.7, B45, B46, B48.7, B50.0, B50.8, B51.0, B51.8, B52.8, B52.0, B55, B57.2-B57.5, B58.0, B59, B67, B69, B73, B74, B78.7, B90-B94, F02.1, K23.1, K93.1, M00, N33.0, P35.0-P35.2, P35.8, P35.9, P37.1
**Respiratory**	Asthma and chronic lower respiratory disease	J41-J47
	Cystic fibrosis	E84, P75
	Injuries	S17, S27, S28, T27, T91.4
	Congenital anomalies	Q30-Q37, Q79.0
	Other	G47.3, J60-J70, J80-J86, J96.1, J98, P27, Y55.6, Z43.0, Z93.0, Z94.2
**Metabolic/endocrine /digestive /renal /genitourinary**	Diabetes	E10-E14, G59.0, G63.2, I79.2, M14.2, N08.3, O24, Y42.3
	Other endocrine	E00, E03.0, E03.1, E07.1, E22.0, E23.0, E25, E26.8, E29.1, E31, E34.1, E34.2, E34.5, E34.8, G13.2, G73.5, Y42.1
	Digestive	K20, K21.0, K22, K23.8, K25-K28, K29.0, K29.1, K29.3-K29.9, K31, K50-K52, K55, K57, K59.2, K63.0-K63.3, K66, K72-K76, K80-K83, K85.0, K85.1, K85.8, K85.9, K86.1-K86.9, K87.0, K90, M07.4, M07.5, M09.1, M09.2, T86.4, Z43.2-Z43.4, Z46.5, Z90.3, Z90.4, Z93.2-Z93.5
	Renal/genitourinary	D63.8, G63.8, G99.8, I68.8, M90.8, N08.4, N00-N05, N07, N11-N15, N16.0, N16.2, N16.4, N16.5, N16.8, N18, N19, N20-N23, N25, N26, N28, N29, N31, N32, N33.8, N35, N36, N39.1, N39.3, N39.4, N40-N42, N70-N74, N80-N82, N85, N86, N87,N88, P96.0, T82.4, T83.1, T83.2, T83.4-T83.9, T85.5, T86.1, Y60.2, Y61.2, Y62.2, Y84.1, Z49, Z93.6, Z94.0, Z99.2
	Congenital anomalies of the digestive/renal/genitourinary system	Q38.0, Q38.3, Q38.4, Q38.6-Q38.8, Q39, Q40.2, Q40.3, Q40.8, Q40.9, Q41, Q42, Q43.1, Q43.3-Q43.7, Q43.9, Q44, Q45, Q50.0, Q51, Q52.0-Q52.2, Q52.4, Q54.0-Q54.3, Q54.8, Q54.9, Q55.0, Q55.5, Q56, Q60.1, Q60.2, Q60.4-Q60.6, Q61, Q62.0-Q62.6, Q62.8, Q63.0-Q63.2, Q63.8, Q63.9, Q64, Q79.2-Q79.5, Q87.8, Q89.1, Q89.2

	Injuries	S36, S37, S38, S39.6, S39.7, T06.5, T28, T91.5
	Other/unspecified	E66, G63.3, G99.0, M14.5, N92, Z86.3, Z93.8
**Musculoskeletal/ skin**	Musculoskeletal/connective tissue	G55.1-G55.3, G63.5, G63.6, G73.7, J99.0, J99.1, L62.0, M05, M06, M07.0-M07.3, M07.6, M08, M09.8, M10-M13, M14.0, M14.6, M14.8, M30-M35, M40-M43, M45-M48,M50-M54, M60-M62, M63.8, M80.1-M80.9, M81.1-M81.9, M82.1, M82.8, M84.0-M84.2, M84.8, M84.9, M85, M86.3-M86.6, M89, M90.0, M91-M94, N08.5, Y45.4
	Skeletal injuries/amputations	S13, S22.0-S22.2, S22.5, S23, S32, S33, S68.3, S68.4, S68.8, S77, S78, S87, S88, S97, S98.0, S98.2-S98.4, T02, T04, T05, T20.3, T20.7, T21.3, T21.7, T22.3, T22.7, T23.2, T23.3, T23.6, T23.7, T24.3, T24.7, T25.2, T25.3, T25.6, T25.7, T29.3, T29.7, T30.3, T30.7, T31.2-T31.9, T32.2- T32.9, T87.3-T87.6, T91.2 T91.8, T92.6, T93.1, T93.4, T93.6, T94.0, T94.1, T95.0, T95.1, T95.4, T95.8, T95.9, Y83.5, Z89.1, Z89.2, Z89.5-Z89.8, Z97.1
	Chronic skin disorders	L10, L11.0, L11.8, L11.9, L12-L14, L28, L40-L45, L57, L58.1, L59, L87, L88, L90, 3 L92, L95, L93, L98.5, M09.0, Q80, Q81, Q87.0-Q87.5, Q89.4
	Congenital anomalies	Q18.8, Q65.0-Q65.2, Q65.8, Q65.9, Q67.5, Q68.2, Q68.3-Q68.5, Q71-Q73, Q74, Q75.3-Q75.9, Q76.1-Q76.4, Q77, Q78, Q79.6, Q79.8, Q82.0-Q82.4, Q82.9, Q86.2, Q89.7-Q89.9
**Neurological**	Epilepsy	F80.3, G40.0-G40.4, G40.6-G40.9, G41, R56.8, Y46.0-Y46.6
	Cerebral palsy	G80-G83
	Injuries of brain, nerves, eyes or ears	S05-S08, S12, S14, S24, S34, S44, S54, S64, S74, S84, S94, T06.0- T06.2, T26, T90.4, T90.5, T91.1, T91.3, T92.4
	Chronic eye conditions	H05.1-H05.9, H13.3, H17, H18, H19.3, H19.8, H21, H26, H27, H28.0-H28.2, H31, H32.8, H33, H34, H35, H40, H42.0, H43, H44, H47, H54.0- H54.2, H54.4, T85.2, T85.3, Z44.2
	Chronic ear conditions	H60.2, H65.2-H65.4, H66.1-H66.3, H69.0, H70.1, H73.1, H74.0-H74.3, H75.0, H80, H81.0, H81.4, H83.0, H83.2, H90.0, H90.3, H90.5, H90.6, H91, Z45.3
	Perinatal conditions	P10, P21.0, P52, P57, P90, P91.1, P91.2, P91.6
	Congenital anomalies of neurological or sensory systems	Q00-Q07, Q10.4, Q10.7, Q11-Q12, Q13.0-Q13.4, Q13.8, Q13.9, Q14-Q16, Q75.0, Q75.1, Q85, Q86.0, Q86.1, Q86.8, Q90-Q93, Q95.2, Q95.3, Q97, Q99
	Other	F02.2, F02.3,G00-G09, G10-G12, G13.8, G14, G20-G23, G24.1-G24.9, G25-G30, G31.0-G31.1, G31.8, G31.9, G32-G37, G43-G46, G47.0-G47.2, G47.4-G47.9, G50- G52,

		G53.0, G53.1, G53.8, G54, G55.8, G56-G58, G59.8, G60, G61, G62.0, G62.2- G62.9, G64, G70, G71,G72.2-G72.9, G73.0, G73.3, G90-G93, G94.2, G94.8, G95, G96, G98, G99.1, G99.2, I60-I67, I68.0, I68.2, I69, I72.0, I72.5, T85.0, T85.1, Y46.7- Y46.8, Z98.2
**Cardiovascular**	Congenital heart disease	Q20-Q26, Q89.3
	Other	I00-I28, I31-I39, I41, I42.0-I42.5, I42.7-I42.9, I43.0, I43.1, I43.2-I43.8, I44.1-I44.7, I45.1-I45.9, I46-I51, I52.8, I70-I71, I72.1-I72.4, I72.8, I72.9, I73-I77, I79.0, I79.1, I79.8, I81-I82, I98-I99, M03.6, N08.8, Q27, Q28, S26, T82.0-T82.3, T82.5-T82.9, T86.2, Y60.5, Y61.5, Y62.5, Y84.0, Z45.0, Z50.0, Z94.1, Z95
**Codes indicating non-specific chronic conditions**	-	R62, R63.3, Z43.1, Z51.5, Z75.5, Z93.1, Z99.3

* Excluding F020-F024 (dementia codes)
** Excluding F51 (nonorganic sleep disorders) and F52 (sexual dysfunction, not caused by organic disorder or disease)
*** Excluding F64 (gender identity disorders (including transsexualism, transvestism, and "gender disorders")

Code lists were derived from the following studies:

- Harron K, Gilbert R, Fagg J, Guttmann A, van der Meulen J. Associations between pre-pregnancy psychosocial risk factors and infant outcomes: a population-based cohort study in England. *The Lancet Public Health* 2021; 6(2): e97-e105.
- Herbert A, Gilbert R, González-Izquierdo A, et al. Violence, self-harm and drug or alcohol misuse in adolescents admitted to hospitals in England for injury: a retrospective cohort study. BMJ Open. 2015;5(2):e006079.
- Hardelid P, Dattani N, Gilbert R. Estimating the prevalence of chronic conditions in children who die in England, Scotland and Wales: a data linkage cohort study. BMJ Open. 2014;4(8).
- Pearson RJ, Jay MA, Wijlaars LPMM, et al. Association between health indicators of maternal adversity and the rate of infant entry to Local Authority care in England: a longitudinal ecological study. *BMJ Open* 2020; 10(8): e036564.

Appendix Table 4: Maternal risk factors prior to enrolment (FNP participants) or antenatal booking appointment (controls) used for propensity score matching and adjustment

Maternal risk factor	Categorisation
Date of delivery	Year / quarter-year
Maternal age at birth	13-15, 16-17, 18-19, 20 years
Ethnicity	White, Black, South Asian, Mixed/Other, or Unknown
Area-level deprivation at birth	Quintile of the Index of Multiple Deprivation
Region of residence	South East, London, North West, East of England, West Midlands, South West, Yorkshire and the Humber, East Midlands, North East
Gestational age at booking	<10 weeks, 10-20 weeks, 20+ weeks
History of hospital attendances in the 2 years before 20 weeks of pregnancy:	Unplanned hospital admissions for adversity-related diagnoses*<br>Unplanned hospital admissions for mental health-related diagnoses*<br>Any hospital admission for chronic condition-related diagnoses*<br>Any A&E attendance<br>Repeated A&E attendance (4+ A&E attendances)<br>Did not attend ≥1 outpatient appointment
History of Social Care contacts before 20 weeks of pregnancy	Ever had a Child Protection Plan<br>Ever a Child Looked After
Educational risk factors before 20 weeks of pregnancy	Ever recorded as having Special Educational Needs provision<br>Ever recorded as having Free School Meals (eligible, applies for and receives)<br>Ever in the most deprived IDACI decile<br>Ever excluded from school, in a pupil referral unit, or alternative provision<br>Ever persistently absent (≥10% of possible sessions)<br>Achieved expected levels at Key Stage 2 Mathematics / English [$]<br>Achieved 5 A*-Cs at GSCE level[^]

* see **Error! Reference source not found.** Table 3; [$] At age 11 years. Only available until 2013/14. [^] Amongst those who were aged ≥16 at the start of the academic year in which they reached 20 weeks of pregnancy; IDACI: Income Deprivation Affecting Children Index

Appendix Table 5: Description of indicators of child maltreatment among study cohort of first-time mothers 13-19 giving birth between April 2010-March 2019 (unadjusted)

	All children in cohort		Children of mothers ever enrolled in FNP		Children of mothers never enrolled in FNP	
	N	%	N	%	N	%
**Total with information on birth outcomes**	**130415**		**31260**		**99150**	
Discharge to social services at birth	630	0.5	255	0.8	375	0.4
**Total with 2 years follow up for health outcomes (Births between April 2010 and March 2017)**	**108675**		**25630**		**83040**	
≥1 Unplanned admission for maltreatment or injury	5790	5.3	1700	6.6	4090	4.9
Mean no. unplanned injury/maltreatment-related admissions[a] (SD)		1.2 (0.7)		1.2 (0.6)		1.2 (0.7)
**Total with 7 years follow up for health outcomes (Births between April 2010 and March 2012)**	**27015**		**4375**		**22640**	
≥1 Unplanned admission for maltreatment or injury	3175	11.8	600	13.7	2575	11.4
Mean no. unplanned injury/maltreatment-related admissions[a] (SD)		1.3 (0.9)		1.3 (0.8)		1.3 (0.9)
**Total with 7 years follow up for social care outcomes (Births between April 2010 and March 2012)**	**17605**		**3250**		**14355**	
Ever Looked After	355	2.0	85	2.6	270	1.9
Mean no. episodes of care [b] (SD)		1.1 (0.3)		1.1 (0.3)		1.1 (0.3)
Ever had a Child Protection Plan	790	4.5	165	5.1	625	4.3
Ever had a CiN referral	3890	22.1	835	25.7	3055	21.3
Mean no. CiN referrals [c] (SD)		1.6 (1.0)		1.6 (1.1)		1.6 (1.0)
Mean no. CiN referrals made by health visitor [c] (SD)		0.0 (0.2)		0.0 (0.2)		0.0 (0.2)

[a] among children with at least one admission; [b] among children with at least one period of care; [c] among children with at least one referral; CiN: Child in Need; SD: Standard deviation; FNP: Family Nurse Partnership

Appendix Table 6: Description of child health outcomes among study cohort of first-time mothers aged 13-19 (unadjusted)

	**All children in cohort**		**Children of mothers ever enrolled in FNP**		**Children of mothers never enrolled in FNP**	
	N	%	N	%	N	%
**Total with information on birth outcomes (Births between April 2010 and March 2019)**	**130415**		**31260**		**99150**	
Total with information on gestational age at birth	121005		28075		92935	
Preterm birth (<37 weeks)	9940	8.2	2650	9.4	7295	7.8
Total with information on birthweight	121815		28350		93460	
Low birthweight (<2,500g)	9395	7.7	2515	8.9	6880	7.4
**Total with 2 years follow up (Births between April 2010 and March 2017)**	**108675**		**25630**		**83040**	
≥1 Unplanned admission (any diagnosis)	40140	36.9	10360	40.4	29780	35.9
Mean no. unplanned admissions[a] (SD)		1.9 (1.9)		1.9 (1.9)		1.9 (1.8)
≥1 A&E attendance	77725	71.5	19570	76.3	58155	70.0
Mean no. A&E attendances[a] (SD)		3.0 (2.8)		3.3 (3.0)		2.9 (2.7)
≥1 Outpatient referral	55630	51.2	14310	55.8	41320	49.8
Mean no. referrals[a]		2.3 (2.6)		2.4 (2.5)		2.3 (2.6)
Did not attend ≥1 outpatient appointment	19745	18.2	5485	21.4	14260	17.2
Mean no. Did not attends[a]		2.0 (1.7)		2.1 (1.9)		2.0 (1.6)
**Total with 7 years follow up (Births between April 2010 and March 2012)**	**27015**		**4375**		**22640**	
≥1 Unplanned admission (any diagnosis)	13195	48.8	2225	50.8	10975	48.5
Mean no. unplanned admissions[a] (SD)		2.3 (3.2)		2.5 (3.8)		2.3 (3.1)
≥1 A&E attendance	23555	87.2	3985	91.0	19570	86.4
Mean no. A&E attendances[a] (SD)		4.9 (4.7)		5.5 (5.3)		4.8 (4.6)
≥1 Outpatient referral	20450	75.7	3460	79.1	16990	75
Mean no. referrals[b]		4.0 (4.3)		4.2 (4.6)		3.9 (4.2)
Did not attend ≥1 outpatient appointment	11150	41.3	2010	45.9	9145	40.4
Mean no. Did not attends[b]		3.0 (3.1)		3.2 (3.5)		2.9 (3.1)

SD: Standard Deviation

[a] among children with at least one admission/attendance/referral/did not attend; Note: numbers have been rounded to the nearest 5 and cell sizes <10 have been supressed, in accordance with NHS Digital's and DfE's statistical disclosure rules for sub-national analyses.

Appendix Table 7: Description of additional child health outcomes among study cohort of first-time mothers aged 13-19 (unadjusted)

	All children in cohort	Children of mothers ever enrolled in FNP	Children of mothers never enrolled in FNP
**Total with 2 years follow up**	108675	25630	83040
Unplanned admissions for any diagnosis			
Mean duration (SD)[a]	2.6 (10.8)	2.8 (11.6)	2.5 (10.6)
Median duration (IQR)	1 (0.5-2)	1 (0.5-2)	1.0 (0.5-2)
N Overnight admissions (%)	24885 (22.9)	6635 (25.9)	18250 (22.0)
N Short stay admissions (%)	25620 (23.6)	2560 (25.6)	19060 (23.0)
Unplanned admissions for maltreatment/injury			
Mean duration (SD)[a]	2.2 (8.6)	1.9 (6.7)	2.3 (9.2)
Median duration (IQR)	1 (0.5-1)	1 (0.5-1)	1 (0.5-1)
N Overnight admissions (%)	3090 (2.8)	935 (3.6)	2155 (2.6)
N Short stay admissions (%)	3095 (2.8)	880 (3.4)	2215 (2.7)
**Total with 7 years follow up**	27015	4375	22640
Unplanned admissions for any diagnosis			
Mean duration (SD)[a]	3.0 (15.6)	3.5 (13.3)	2.9 (16.0)
Median duration (IQR)	1 (0.5-2)	1 (0.5-3)	1 (0.5-2)
N Overnight admissions (%)	8510 (31.5)	1505 (34.4)	7005 (30.9)
N Short stay admissions (%)	9035 (33.4)	1505 (34.4)	7530 (33.3)
Unplanned admissions for maltreatment/injury			
Mean duration (SD)[a]	2.0 (13.5)	2.2 (10.3)	2.0 (14.1)
Median duration (IQR)	1 (0.5-1)	1 (0.5-1)	1 (0.5-1)
N Overnight admissions (%)	1635 (6.1)	315 (7.2)	1315 (5.8)
N Short stay admissions (%)	1855 (6.9)	340 (7.8)	1515 (6.7)

SD: Standard Deviation. IQR: Interquartile range

[a] among children with at least one admission/attendance/referral/did not attend; Note: numbers have been rounded to the nearest 5 and cell sizes <10 have been supressed, in accordance with NHS Digital's and DfE's statistical disclosure rules for sub-national analyses.

Appendix Table 8: Description of child developmental and educational outcomes among study cohort of first-time mothers aged 13-19 (unadjusted)

	All children in cohort		Children of mothers ever enrolled in FNP		Children of mothers never enrolled in FNP	
	N	%	N	%	N	%
**Total with information on nursery attendance**	**25140**		**4135**		**21010**	
Attended nursery between ages 2-4	24090	95.8	3955	90.4	20135	95.8
**Total with information on school readiness at age 5 (EYFSP)**	**24585**		**4035**		**20545**	
Good Level of Development (across all 5 domains)	14445	58.5	2325	53.1	12120	59.0
GLD: Communication and Language	18595	75.6	3010	74.6	15585	75.9
GLD: Physical Development	20340	82.7	3325	82.4	17010	82.8
GLD: Personal, Social and Emotional Development	19345	78.7	3130	77.6	16215	78.9
GLD: Literacy	15090	61.4	2435	60.3	12655	61.6
GLD: Maths	16630	67.6	2685	66.5	13945	67.9
**Total with information at Key Stage 1**	**24530**		**4040**		**20490**	
Expected level of development at KS1 (Maths)	16015	65.3	2580	63.9	13435	65.6
Expected level of development at KS1 (Writing)	14215	57.9	2255	55.9	11960	58.4
Expected level of development at KS1 (Reading)	16255	66.3	2635	65.3	13620	66.5
**Total with information on Special Educational Needs provision and Free School Meals**	**24925**		**4105**		**20820**	
Ever recorded as having Special Educational Needs	6175	24.8	1120	27.8	5060	24.3
Ever recorded as having Free School Meals	11780	47.3	2290	56.8	9485	45.6
**Total with information on Persistent Absence**	**25155**		**4135**		**21020**	
Ever persistently absent	14700	58.4	2555	63.3	12145	57.8

EYFSP: Early Years Foundation Stage Profile; GLD: Good Level of Development; KS: Key Stage

Note: numbers have been rounded to the nearest 5 and cell sizes <10 have been supressed, in accordance with NHS Digital's and DfE's statistical disclosure rules for sub-national analyses.

Appendix Table 9: Description of maternal outcomes among study cohort of first-time mothers aged 13-19 (unadjusted)

	All mothers in cohort		Mothers enrolled in FNP		Mothers never enrolled in FNP	
	N with outcome	%	N with outcome	%	N with outcome	%
**Total with 2 years follow up for health outcomes (Deliveries between April 2010 and March 2017)**	**110555**		**25690**		**84860**	
≥1 Unplanned admission for adversity-related diagnoses	1890	1.7	695	2.7	1195	1.4
Mean no. unplanned adversity-related admissions[a] (SD)		1.3 (1.1)		1.4 (1.2)		1.3 (1.1)
≥1 Unplanned admission for mental health-related diagnoses (excluding substance misuse and self-harm)	2900	2.6	1075	4.2	1825	2.1
Mean no. unplanned mental health-related admissions [a] (SD)		1.5 (1.5)		1.6 (1.5)		1.5 (1.4)
Unplanned admission for any diagnosis	18975	17.2	5210	20.3	13765	16.2
Mean no. unplanned admissions [a] (SD)		1.6 (1.4)		1.7 (1.6)		1.6 (1.3)
≥1 A&E attendance	54700	49.5	14470	56.3	40230	47.4
Mean no. A&E attendances [a] (SD)		2.5 (2.7)		2.8 (3.3)		2.3 (2.5)
Subsequent delivery within 18 months	10230	8.8	2325	8.5	7905	8.9
**Total with information on educational attainment [b]**	**8145**		**4225**		**3915**	
Mother achieved 5 A*-C inc. Eng/Maths at KS4	820	10.1	405	9.6	415	10.6
**Total with information on school enrolment up to Year 11 [c]**	2035		4290		18800	
School enrolment	1675	82.3	960	81.8	710	82.9
**Total with 7 years follow up for health outcomes (Deliveries between April 2010 and March 2012)**	**27250**		**4385**		**22865**	
≥1 Unplanned admission for adversity-related diagnoses	1535	5.6	345	7.9	1190	5.2
Mean no. unplanned adversity-related admissions [a] (SD)		1.6 (1.6)		1.7 (2.0)		1.5 (1.4)
≥1 Unplanned admission for mental health-related diagnoses (excluding substance misuse and self-harm)	2095	7.7	440	10.0	1655	7.2
Mean no. unplanned mental health-related admissions [a] (SD)		1.9 (2.4)		2.1 (3.0)		1.9 (2.1)
Unplanned admission for any diagnosis	11585	42.5	2090	47.7	9495	41.5
Mean no. unplanned admissions [a] (SD)		2.4 (2.9)		2.5 (3.2)		2.3 (2.8)
≥1 A&E attendance	22065	81.0	3790	86.5	18275	79.9
Mean no. A&E attendances [a] (SD)		5.2 (6.7)		6.3 (8.5)		5.0 (6.2)

[a] among mothers with at least one admission/attendance; [b] among mothers who were <16 at the start of the academic year in which they reached 20 weeks of pregnancy; [c] up to Year 11, among mothers who were <15 at the start of the academic year in which they reached 20 weeks of pregnancy

Note: numbers have been rounded to the nearest 5 and cell sizes <10 have been supressed, in accordance with NHS Digital's and DfE's statistical disclosure rules for sub-national analyses.

A&E; Accident & Emergency; SD: Standard Deviation; KS: Key Stage

Appendix Figure 2: Overlap in the distribution of propensity scores between mothers who were and were not enrolled in FNP

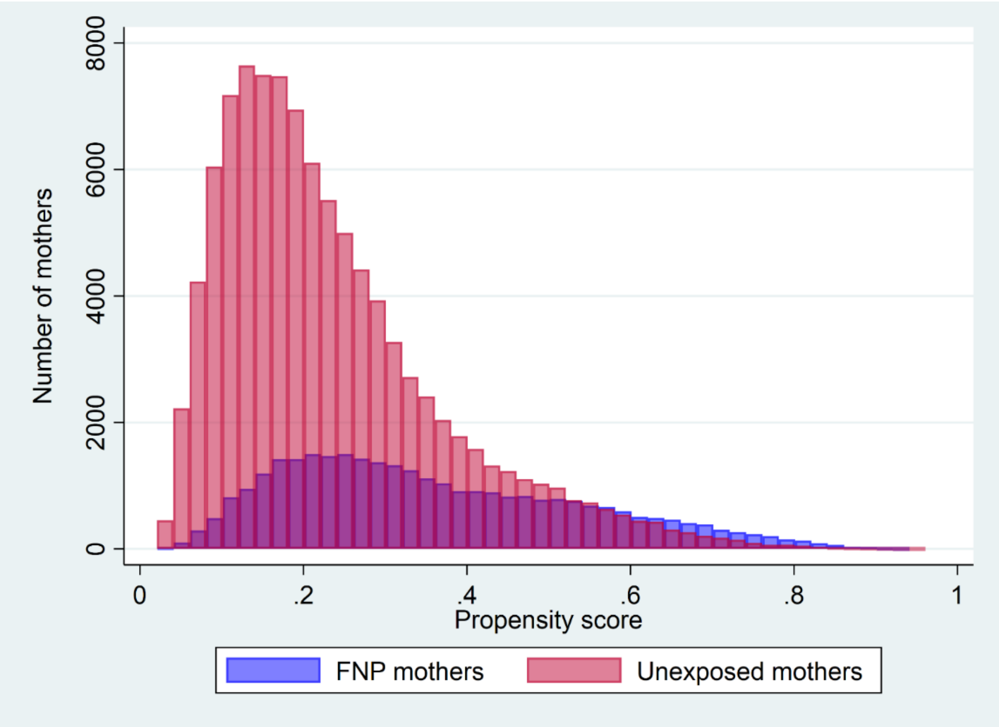

Appendix Figure 3: Standardised differences comparing maternal risk factors in the propensity-score matched cohort of all mothers aged 13-19 giving birth between April 2010 and March 2019.

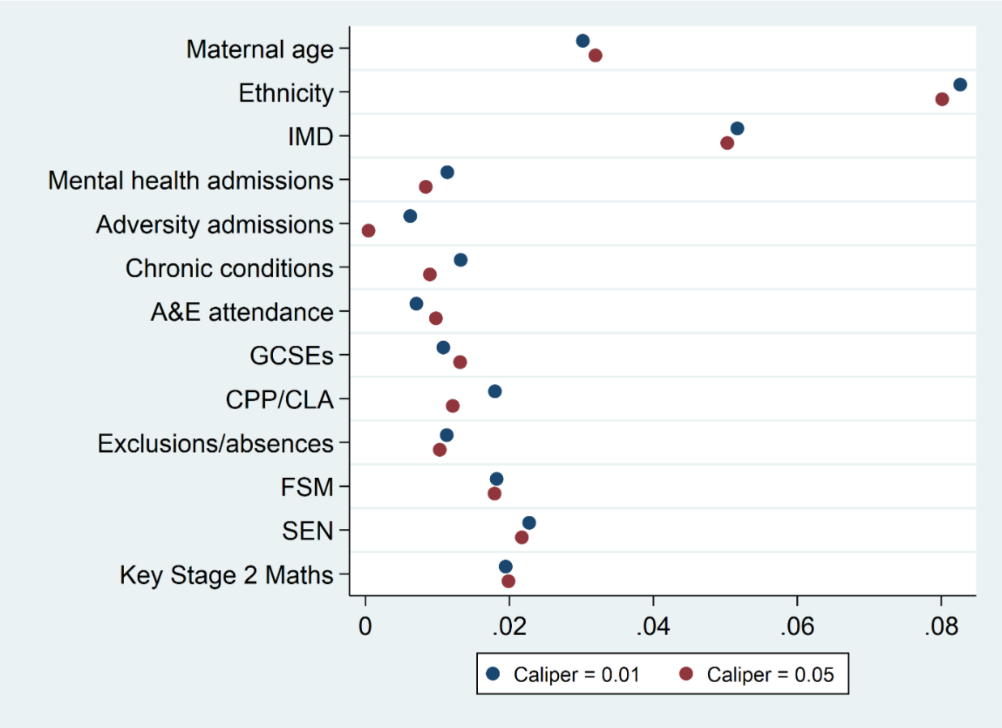

Note: The final matched cohort used a caliper width of 0.01. Standardised differences of 0.2, 0.5 and 0.8 are considered to be small, medium and large effect sizes respectively.

Appendix Table 10: Relative risks and 95% Confidence Intervals for indicators of child maltreatment comparing mothers enrolled in the FNP versus mothers who were not enrolled, for mothers aged 13-19 in the propensity score matched cohort

	N (%) in mothers enrolled in FNP (treated)	N (%) in mothers never enrolled in FNP (untreated)	Adjusted Relative Risk (95% CI)
**Birth outcomes (Births between April 2010 and March 2019)**			
Total with information at discharge	*28995*	*28710*	
Discharge to social services	215 (0.7)	180 (0.6)	1.23 (1.00, 1.51)
**Child outcomes - within 2 years (Births between April 2010 and March 2017)**			
Total with information on health outcomes within 2 years	*24240*	*23790*	
≥ 1 Unplanned admission for maltreatment or injury	1605 (6.6)	1385 (5.7)	1.15 (1.07, 1.24)
**Child outcomes - within 7 years Births between April 2010 and March 2012**			
Total with information on health outcomes within 7 years	*4330*	*4310*	
≥ 1 Unplanned admission for maltreatment or injury	595 (13.6)	575 (13.2)	1.03 (0.93, 1.14)
Total with information on social care outcomes within 7 years	*3215*	*2965*	
Child Looked After	85 (2.6)	85 (2.9)	0.91 (0.68, 1.21)
Child Protection Plan	165 (5.1)	180 (6.1)	0.84 (0.71, 1.00)
Child in Need referral	830 (19.0)	785 (18.0)	0.99 (0.91, 1.07)

Note: numbers have been rounded to the nearest 5 and cell sizes <10 have been supressed, in accordance with NHS Digital's and DfE's statistical disclosure rules for sub-national analyses.

Appendix Table 11: Relative risks and 95% Confidence Intervals for health outcomes comparing mothers enrolled in the FNP versus mothers who were not enrolled, for mothers aged 13-19 in the propensity score matched cohort

	N (%) in mothers enrolled in FNP (treated)	N (%) in mothers never enrolled in FNP (untreated)	Adjusted Relative Risk (95% CI)
**Birth outcomes (Births between April 2010 and March 2019)**			
Total with information on gestational age at birth	*26485*	*27375*	
Preterm birth (<37 weeks of gestation)	2465 (8.4)	1375 (8.0)	1.04 (0.99, 1.00)
Total with information on birth weight	*26740*	*27625*	
Low birth weight (<2500g)	2345 (7.9)	2240 (7.6)	1.07 (1.02, 1.13)
**Child health outcomes - within 2 years (Births between April 2010 and March 2017)**			
Total with information on health outcomes within 2 years	*24240*	*23790*	
≥ 1 Unplanned admission for any diagnosis	9705 (39.7)	9110 (37.3)	1.06 (1.03, 1.09)
≥ 1 A&E attendance	13435 (54.9)	12235 (50.0)	1.04 (1.03, 1.05)
≥ 1 Outpatient referral	5110 (21.1)	4455 (18.7)	1.10 (1.07, 1.12)
Did not attend ≥ 1 outpatient appointment	1990 (46.0)	1860 (43.2)	1.11 (1.06, 1.15)
**Child health outcomes - within 7 years (Births between April 2010 and March 2012)**			
Total with information on health outcomes within 7 years	*4330*	*4310*	
≥ 1 Unplanned admission for any diagnosis	2200 (50.5)	2225 (51.0)	1.01 (0.96, 1.05)
≥ 1 A&E attendance	3945 (90.5)	3800 (87.2)	1.03 (1.02, 1.05)
≥ 1 Outpatient referral	18460 (75.5)	17285 (70.7)	1.05 (1.02, 1.08)
Did not attend ≥ 1 outpatient appointment	3430 (78.7)	3265 (74.9)	1.07 (1.01, 1.12)

GLD: A&E: Accident & Emergency
Note: numbers have been rounded to the nearest 5 and cell sizes <10 have been supressed, in accordance with NHS Digital’s and DfE’s statistical disclosure rules for sub-national analyses.

Appendix Table 12: Relative risks and 95% Confidence Intervals for additional child health outcomes comparing mothers enrolled in the FNP versus mothers who were not enrolled, for mothers aged 13-19 in the propensity score matched cohort

	N (%) in mothers enrolled in FNP	N (%) in mothers not enrolled in FNP	Adjusted relative risk (95% CI)
**Child health outcomes - within 2 years (Births between April 2010 and March 2017)**			
Total with information on health outcomes within 2 years	24240	23790	
All unplanned admissions			
Overnight admissions	6200 (25.6)	5620 (23.6)	1.10 (1.07, 1.14)
Short stay admissions	6140 (25.3)	5880 (24.7)	1.21 (1.10, 1.33)
Unplanned admissions for maltreatment/injury			
Overnight admissions	875 (3.6)	725 (3.0)	1.04 (1.01, 1.08)
Short stay admissions	1015 (4.2)	760 (3.2)	1.31 (1.15, 1.48)
**Child health outcomes - within 7 years (Births between April 2010 and March 2012)**			
Total with information on health outcomes within 7 years	4330	4310	
All unplanned admissions			
Overnight admissions	1485 (34.3)	1430 (33.2)	1.05 (0.98, 1.12)
Short stay admissions	1485 (34.3)	1540 (35.7)	1.05 (0.90, 1.22)
Unplanned admissions for maltreatment/injury			
Overnight admissions	310 (7.2)	300 (7.0)	0.99 (0.93, 1.06)
Short stay admissions	365 (8.4)	330 (7.7)	1.11 (0.97, 1.29)

Note: numbers have been rounded to the nearest 5 and cell sizes <10 have been supressed, in accordance with NHS Digital's and DfE's statistical disclosure rules for sub-national analyses.

Appendix Table 13: Relative risks and 95% Confidence Intervals for child developmental and educational outcomes comparing mothers enrolled in the FNP versus mothers who were not enrolled, for mothers aged 13-19 in the propensity score matched cohort

	N (%) in mothers enrolled in FNP (treated)	N (%) in mothers never enrolled in FNP (untreated)	Adjusted Relative Risk (95% CI)
**Child developmental/educational outcomes - within 7 years (Births between April 2010 and March 2012)**			
Total with information on nursery attendance	*4090*	*4040*	
Nursery attendance	3915 (89.8)	3870 (88.8)	1.00 (0.99, 1.01)
Total with information on EYFSP	*3990*	*3955*	
Good Level of Development (school readiness)	2295 (57.5)	2190 (55.4)	1.05 (1.00, 1.09)
GLD: Communication and Language	2980 (74.7)	2930 (74.1)	1.01 (0.98, 1.04)
GLD: Physical Development	3290 (82.5)	3220 (81.4)	1.01 (0.99, 1.04)
GLD: Personal, Social and Emotional Development	3095 (77.6)	3040 (76.9)	1.01 (0.99, 1.03)
GLD: Literacy	2400 (60.2)	2290 (57.9)	1.04 (1.00, 1.08)
GLD: Maths	2655 (66.5)	2570 (65.0)	1.03 (0.99, 1.07)
Total with information on Key Stage 1 attainment	*4270*	*4260*	
Expected levels at KS1 (maths)	2550 (58.5)	2485 (57.0)	0.98 (0.95, 1.02)
Expected levels at KS1 (reading)	2600 (59.6)	2490 (57.1)	1.00 (0.96, 1.04)
Expected levels at KS1 (writing)	2225 (51.0)	2180 (50.0)	0.97 (0.93, 1.02)
Total with information on SEN / FSM	*4060*	*4010*	
Special Educational Needs provision	1105 (25.3)	1025 (23.5)	1.06 (0.99, 1.14)
Free school meals	2265 (51.9)	2035 (46.7)	1.09 (1.04, 1.14)
Total with information on absence	*4090*	*4040*	
Persistent absence	2530 (61.9)	2450 (60.6)	1.01 (0.97, 1.04)

GLD: Good Level of Development; EYFSP: Early Years Foundation Stage Profile; KS: Key Stage; SEN: Special Educational Needs; FSM: Free School Meals

Note: numbers have been rounded to the nearest 5 and cell sizes <10 have been supressed, in accordance with NHS Digital’s and DfE’s statistical disclosure rules for sub-national analyses.

Appendix Table 14: Relative risks and 95% Confidence Intervals for maternal outcomes comparing mothers enrolled in the FNP versus mothers who were not enrolled, for mothers aged 13-19 in the propensity score matched cohort

	N (%) in mothers enrolled in FNP (treated)	N (%) in mothers never enrolled in FNP (untreated)	Adjusted Relative Risk (95% CI)
**Maternal outcomes - 2 years**<br>**Births between April 2010 and March 2017**			
Total with information on health outcomes within 2 years	*24455*	*24455*	
≥1 Unplanned admission for adversity-related diagnoses	605 (2.5)	485 (2.0)	1.27 (1.15, 1.41)
≥1 Unplanned admission for mental health related diagnoses	950 (3.9)	745 (3.0)	1.29 (1.18, 1.41)
≥1 Unplanned admission for any diagnosis	4860 (19.9)	4520 (18.5)	1.08 (1.04, 1.12)
≥1 A&E attendance	13610 (55.7)	12750 (52.1)	1.06 (1.04, 1.08)
Subsequent delivery within 18 months	2065 (8.4)	2270 (9.3)	0.92 (0.88, 0.97)
Total eligible for and with information on GCSEs	2825	2765	
5 A*-Cs at GCSE level	330 (11.7)	255 (9.2)	1.12 (0.96, 1.29)
Total eligible for and with information on school enrolment	770	680	
School enrolment	620 (80.5)	570 (83.8)	0.96 (0.92, 1.01)
**Maternal outcomes - 7 years**<br>**Births between April 2010 and March 2012**			
Total with information on health outcomes within 7 years	*4360*	*4360*	
≥1 Unplanned admissions for adversity-related diagnoses	345 (7.9)	300 (6.9)	1.16 (1.03, 1.30)
≥1 Unplanned admission for mental health related diagnoses	435 (10.0)	385 (8.8)	1.18 (1.04, 1.33)
≥1 Unplanned admission for any diagnosis	2080 (47.7)	2040 (46.8)	1.02 (0.98, 1.06)
≥1 A&E attendance	3775 (86.6)	3665 (84.1)	1.03 (1.01, 1.05)

A&E: Accident & Emergency
Note: numbers have been rounded to the nearest 5 and cell sizes <10 have been supressed, in accordance with NHS Digital's and DfE's statistical disclosure rules for sub-national analyses.

Appendix Figure 4. Child unplanned admissions for maltreatment/injury in the 2 years following birth: Subgroup analysis presenting adjusted relative risks and 95% Confidence Intervals comparing mothers enrolled in the FNP versus mothers who were not enrolled, for mothers aged 13-19 and giving birth in an area in which FNP was offered at the time of pregnancy, in the propensity score matched cohort.

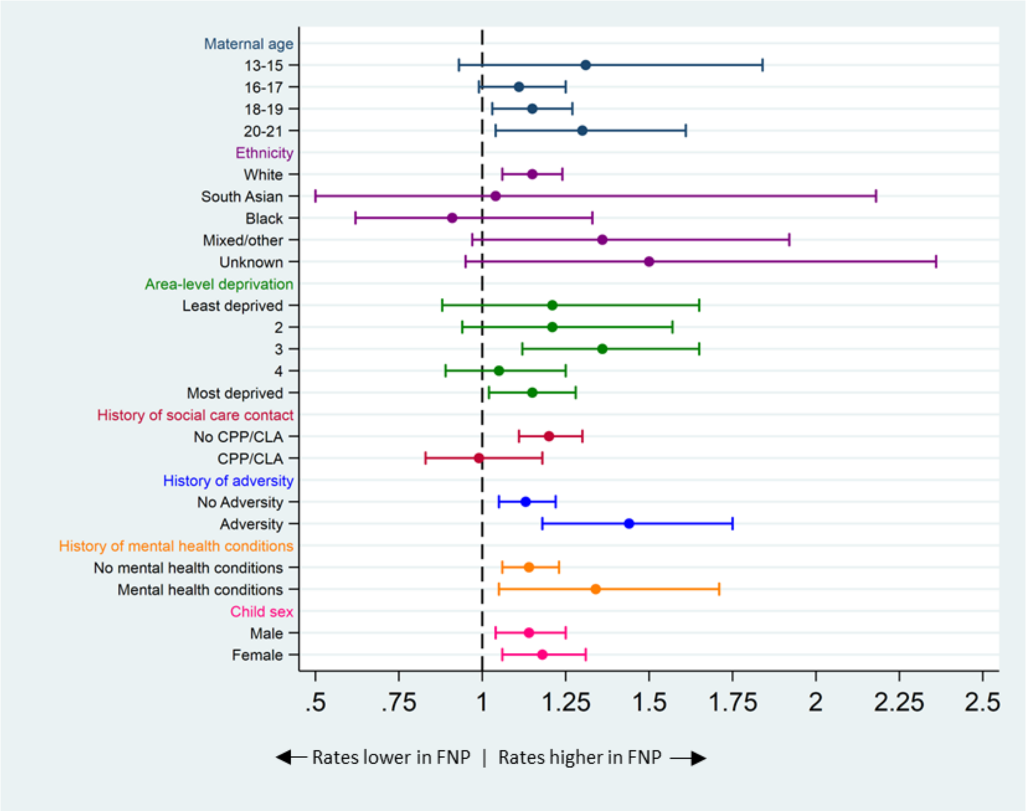

Appendix Figure 5: Good level of Development: Subgroup analysis presenting adjusted relative risks and 95% Confidence Intervals comparing mothers enrolled in the FNP versus mothers who were not enrolled, for mothers aged 13-19 and giving birth in an area in which FNP was offered at the time of pregnancy, in the propensity score matched cohort.

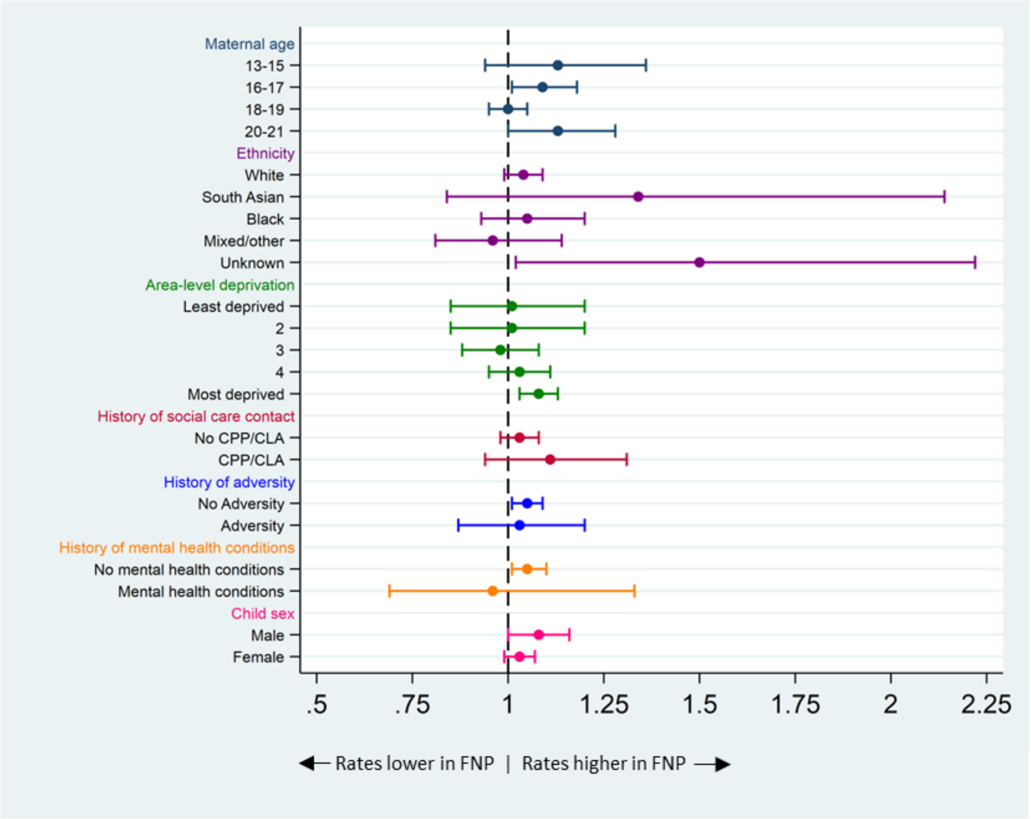

Appendix Figure 6: Subsequent delivery within 18 months: Subgroup analysis presenting adjusted relative risks and 95% Confidence Intervals comparing mothers enrolled in the FNP versus mothers who were not enrolled, for mothers aged 13-19 and giving birth in an area in which FNP was offered at the time of pregnancy, in the propensity score matched cohort.

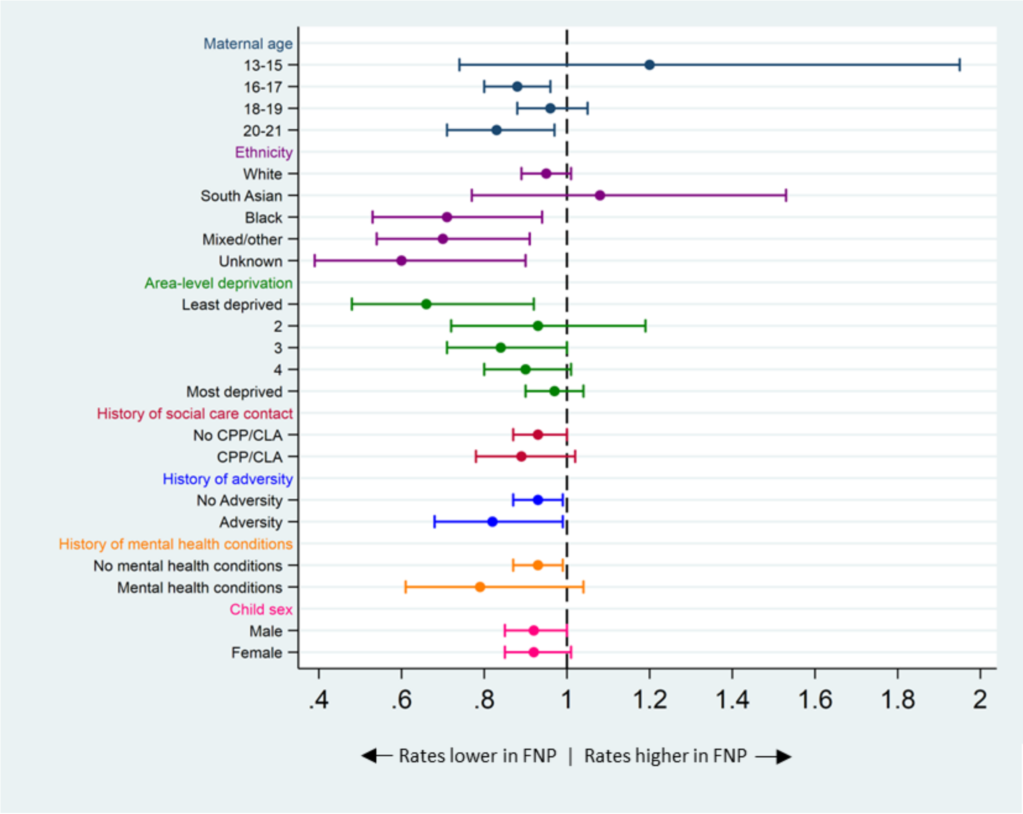

Appendix Figure 7: Maternal unplanned admissions for any diagnosis in the 2 years following delivery: Subgroup analysis presenting adjusted relative risks and 95% Confidence Intervals comparing mothers enrolled in the FNP versus mothers who were not enrolled, for mothers aged 13-19 and giving birth in an area in which FNP was offered at the time of pregnancy, in the propensity score matched cohort.

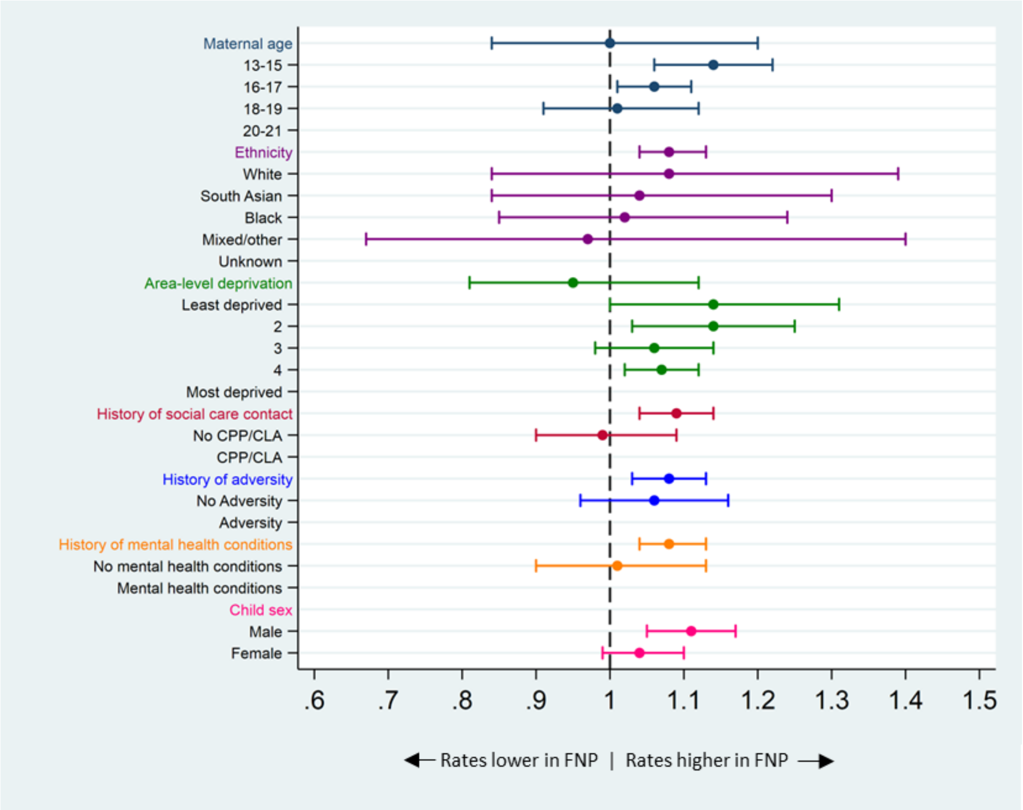

Supplement: online supplemental file 1 [file bmjph-2-1-s001.pdf]
